# Reduced Selective Constraint in Endosymbionts: Elevation in Radical Amino Acid Replacements Occurs Genome-Wide

**DOI:** 10.1371/journal.pone.0028905

**Published:** 2011-12-14

**Authors:** Jennifer J. Wernegreen

**Affiliations:** 1 Nicholas School of the Environment, Duke University, Durham, North Carolina, United States of America; 2 Institute for Genome Sciences and Policy, Duke University, Durham, North Carolina, United States of America; Michigan State University, United States of America

## Abstract

As predicted by the nearly neutral model of evolution, numerous studies have shown that reduced N_e_ accelerates the accumulation of slightly deleterious changes under genetic drift. While such studies have mostly focused on eukaryotes, bacteria also offer excellent models to explore the effects of N_e_. Most notably, the genomes of host-dependent bacteria with small N_e_ show signatures of genetic drift, including elevated K_a_/K_s_. Here, I explore the utility of an alternative measure of selective constraint: the per-site rate of radical and conservative amino acid substitutions (D_r_/D_c_). I test the hypothesis that purifying selection against radical amino acid changes is less effective in two insect endosymbiont groups (*Blochmannia* of ants and *Buchnera* of aphids), compared to related gamma-Proteobacteria. Genome comparisons demonstrate a significant elevation in D_r_/D_c_ in endosymbionts that affects the majority (66–79%) of shared orthologs examined. The elevation of D_r_/D_c_ in endosymbionts affects all functional categories examined. Simulations indicate that D_r_/D_c_ estimates are sensitive to codon frequencies and mutational parameters; however, estimation biases occur in the opposite direction as the patterns observed in genome comparisons, thereby making the inference of elevated D_r_/D_c_ more conservative. Increased D_r_/D_c_ and other signatures of genome degradation in endosymbionts are consistent with strong effects of genetic drift in their small populations, as well as linkage to selected sites in these asexual bacteria. While relaxed selection against radical substitutions may contribute, genome-wide processes such as genetic drift and linkage best explain the pervasive elevation in D_r_/D_c_ across diverse functional categories that include basic cellular processes. Although the current study focuses on a few bacterial lineages, it suggests D_r_/D_c_ is a useful gauge of selective constraint and may provide a valuable alternative to K_a_/K_s_ when high sequence divergences preclude estimates of K_s_. Broader application of D_r_/D_c_ will benefit from approaches less prone to estimation biases.

## Introduction

### Evolutionary significance of N_e_

In considering the evolutionary fate of a new mutation, a critical parameter is the product of the selection coefficient (*s*) for or against that mutation and effective population size (N_e_), which dictates the efficacy of selection. Selection determines the fate of mutations that are strongly deleterious or advantageous (|N_e_ *s*|≫10), and mutations with negligible fitness effects (|N_e_ *s*|≪1) should behave neutrally. However, for a narrow range between these values, the fate of mutations will depend on a balance between selection and the stochastic effect of genetic drift. Ohta [1], [2] emphasized that many mutations fall into this ‘nearly neutral’ category, with selection coefficients near the reciprocal of N_e_. In general, a random mutation is more likely to be deleterious than beneficial [3], [4]. Ohta proposed that the fate of mildly deleterious mutations depends on N_e_. Namely, when N_e_ is reduced, genetic drift plays a greater role and purifying selection against such mutations is less effective. This theory generated a key prediction: reduced N_e_ will lead to the greater accumulation of deleterious changes.

Given the biological significance of slightly deleterious mutations, it is not surprising that much effort has centered on quantifying their abundance and understanding the factors that govern their fate in natural populations. Deleterious mutations likely influence a wide range of biological phenomena, including inbreeding depression, mate choice, Y chromosome and mitochondrial genome degradation, and the maintenance of variation in fitness (reviewed in [5]). Major evolutionary transitions, such as the emergence of sex and recombination, may be mechanisms to avoid the accumulation of such changes [6]–[10]. Their persistence may also influence the demographic fate of small populations [11].

As predicted by the nearly neutral theory, empirical studies have documented a greater accumulation of deleterious changes under genetic drift in small populations. This work spans diverse taxonomic groups, including vertebrates (e.g., rodents, primates and birds), invertebrates, plants, fungi, and bacteria. Evidence supporting the importance of N_e_ includes faster rates of sequence evolution and apparent gene degradation in species with relatively small N_e_ [12]–[18] or short generation times, which may correspond to small populations [19], [20]. Consistent with this trend, island species often show greater signatures of deleterious changes than do mainland relatives [21]–[24]. Moreover, observations that mitochondrial datasets often show deviations from neutrality may reflect the lower N_e_ of haploid, maternally inherited genomes [25]–[27].

In addition to drift-based dynamics in small populations, genetic linkage can also influence the fate of deleterious changes. Species with little or no recombination, such as asexual species, may lack the ability to purge deleterious mutations, a phenomenon known as Muller's ratchet [8]. Similarly, in regions of low recombination, linkage to selected sites may contribute to deleterious substitutions. For instance, selection against strongly deleterious mutations reduces variation at linked sites and can speed the fixation of slightly deleterious mutations [28], [29]. Hitchhiking with selective sweeps of beneficial mutations may also drive the fixation of neutral and even deleterious changes at linked sites [30]. In fact, this contribution of selective sweeps to deleterious evolution, termed genetic *draft*, may be more significant than genetic drift when recombination is low [31]–[34]. Such linkage during selective sweeps has likely contributed to the degradation of Y chromosomes [35] and perhaps mitochondria [34], as well as genomes of organisms under strong artificial selection during the process of domestication, including certain crops [36].

While most studies of deleterious evolution have focused on eukaryotic groups, bacteria also offer promising models to explore the significance of deleterious mutations and processes that influence their dynamics. In particular, the transition from a free-living existence to an obligate host association can profoundly influence N_e_ [18], [37]–[39]. By virtue of their constrained lifestyle and the potential for severe bottlenecks upon transmission to new hosts, host-dependent bacteria are thought to have much smaller effective population sizes than related bacterial lineages that can replicate independently [40]–[42]. Host-dependent species include several well-known bacterial pathogens, as well as long-term intracellular mutualists that are critical to the reproduction and success of many insect species [43]. As extreme examples of host specialization, many such mutualists have coevolved with a particular insect group for tens to hundreds of millions of years. The near absence of recombination in these mutualists may exacerbate the effects of genetic drift due to Muller's ratchet [17] and linkage to selected sites (see above). Abundant evidence points to increased accumulation of deleterious changes in these long-term endosymbionts (reviewed in [44]) and other host-dependent bacterial species [37], [45]. This evidence includes a genome-wide acceleration of evolutionary rates that is concentrated at nonsynonymous sites, thus elevating the ratio of nonsynonymous to synonymous substitutions (K_a_/K_s_ and similar measures). In addition, population genetic analyses have revealed patterns that point to reduced N_e_, such as exceptionally low levels of intraspecific polymorphism, an excess of nonsynonymous polymorphisms, and excess of rare alleles [41], [42]. These findings indicate that evolutionary models developed in eukaryotic taxa have predictive power when applied to bacteria.

### Measuring selective constraint

Empirical tests of the nearly neutral model often compare the effects of purifying selection in species or gene regions with relatively large versus small N_e_, predicting lower selective constraint in the latter. Selective constraint is typically estimated by K_a_/K_s_, which offers a gauge for the effects of purifying selection on protein divergence. This use of K_a_/K_s_ assumes that nonsynonymous mutations have greater fitness consequences than do synonymous changes, and that protein-altering mutations are more likely to be deleterious than beneficial. On this basis, K_a_/K_s_ will be suppressed to the extent that purifying selection effectively removes nonsynonymous mutations. Conversely, elevated K_a_/K_s_ suggests that purifying selection is relaxed due to reduction of selection coefficients, or less effective due to a greater influence of genetic drift. While relaxed selection is expected to affect particular loci or functional categories, reduced efficacy of selection under genetic drift is expected to elevate K_a_/K_s_ across the genome [45]–[47]. Positive, diversifying selection at a particular gene can also elevate K_a_/K_s_, but this force is expected to be relatively rare. K_a_/K_s_ has many attributes as a metric of purifying selection, but it is limited to close sequence comparisons for which K_s_ can be estimated reliably.

For more distant sequence comparisons, the relative abundances of radical and conservative amino acid changes may offer an alternative measure of selective constraint. Several classifications exist to categorize amino acid substitutions based on changes in physiochemical properties such as charge, polarity, and/or volume. While positive selection may favor radical amino acid changes at particular genes (but see [48], [49]), most radical changes are expected to be deleterious and therefore subjected to purifying rather than positive selection. The prediction that radical changes experience stronger purifying selection than conservative changes is supported by the far greater abundance of conservative changes in most proteins.

The ratio of per-site rates of radical and conservative amino acid substitutions (D_r_/D_c_) has been used as a measure of selective constraint, analogous to K_a_/K_s_, and is potentially valuable for measuring the strength or efficacy of purifying selection [4], [16], [36], [48], [50]–[52]. An advantage of D_r_/D_c_ is its utility for species pairs in which high synonymous divergence confounds estimates of K_a_/K_s_. With some exceptions (e.g., [50]), several studies show elevated D_r_/D_c_ in small populations [4], [16], [48], [52], a pattern consistent with reduced efficacy of purifying selection against radical amino acid changes.

In the present study, I explore the utility of D_r_/D_c_ to assess selective constraint in bacteria. Although several studies of eukaryotic taxa have employed this ratio, to my knowledge this is the first study exploring its utility for studying bacterial evolution. Specifically, I test the hypothesis that purifying selection against radical amino acid changes is less effective in bacteria with reduced N_e_. To this end, I track patterns of protein divergence within genome pairs that span ten bacterial lineages. The endosymbionts include *Blochmannia* and *Buchnera*, obligate intracellular mutualists of ants and aphids, respectively. Non-endosymbiotic species include the related enteric bacteria *Escherichia coli* and *Salmonella enterica* serovar Typhimurium (called *Salmonella typhimurium* here), and the more distantly related gamma-Proteobacteria *Shewanella* sp., *Acinetobacter* sp., and *Pseudomonas putida*. Although the non-endosymbiotic species can be host-associated, they do not rely on hosts for reproduction.

However promising, D_r_/D_c_ is not without controversy. For example, its use for detecting positive selection is questionable [49]. Although I use D_r_/D_c_ to measure the effects of purifying (not positive) selection, I must still consider that mutational parameters and base composition can influence estimates of D_r_ and D_c_ and their ratio [48], [49]. Compared to K_a_/K_s_, far less effort has been devoted to mitigating estimation biases for D_r_/D_c_. Smith [48] found that sequence composition can influence D_r_/D_c_ and developed modifications implemented by in-house scripts to reduce that bias; however, the modifications are not readily accessible. In the current study, I use simulations to evaluate estimation biases and to confirm that they cannot explain patterns observed in genome comparisons.

As detailed below, both insect endosymbionts show a consistent increase in D_r_/D_c_ compared to their free-living relatives. Simulations confirm a significant effect of base composition on D_r_/D_c_ but in the opposite direction of the patterns observed from genome comparisons, thereby making the D_r_/D_c_ comparisons more conservative. The persistent elevation of D_r_/D_c_ in endosymbionts further supports reduced selective constraint in these species. While other explanations exist, I argue this result is best explained by reduced efficacy of purifying selection against radical amino acid changes in bacteria with small N_e_, as predicted by the nearly neutral theory. Although I focused on a few bacterial lineages, this study offers proof in concept that D_r_/D_c_ is useful for assessing selective constraint and may offer a valuable tool when high sequence divergences preclude reliable estimates of K_s_. Broader application of D_r_/D_c_ to diverse bacterial genomes would benefit from improved estimation algorithms so that values are less sensitive to base composition and mutational parameters.

## Results

### Pairwise genome divergence summaries

**Figure 1:**
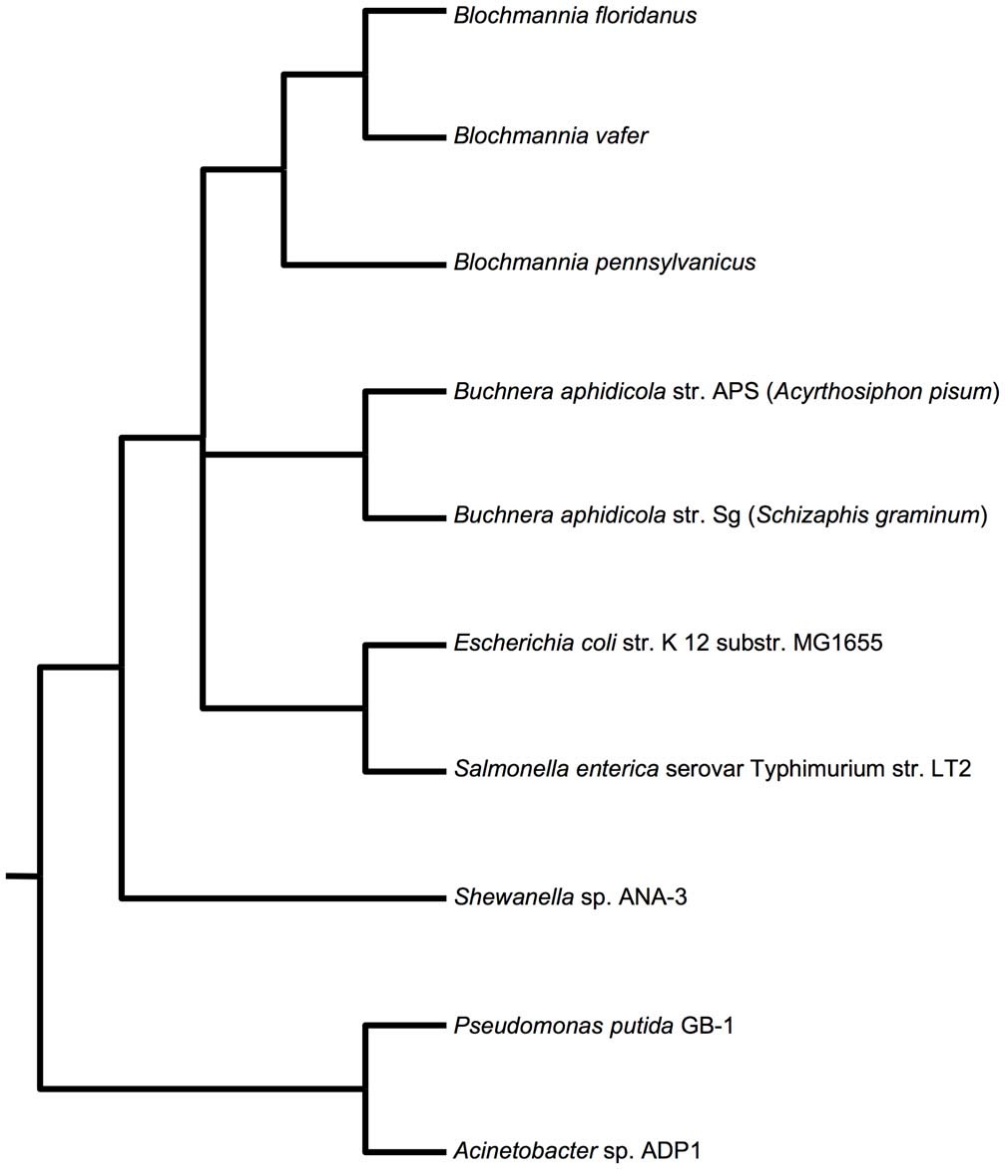
Schematic tree of the bacterial genomes included in comparisons. *Blochmannia* and *Buchnera* have coevolved with their insect hosts, and sequence divergence within each pair reflects changes that have occurred in the context of their endosymbiotic lifestyle. The six pairwise comparisons made across these bacterial lineages are listed in Table 2.

Schematic relationships among the ten genomes compared are shown in Figure 1 and genome information listed in Table 1. The broader phylogenetic position of such AT-rich endosymbionts is difficult to resolve and is left ambiguous in this tree. However, a recent study of the gamma-Proteobacteria [53] suggests that *Blochmannia* and *Buchnera* represent two independent origins of endosymbiosis. Because *Blochmannia* and *Buchnera* have coevolved with their respective insect hosts, sequence divergence within each reflects changes that have occurred in the context of an endosymbiotic lifestyle. Taxa for pairwise comparisons were selected to include phylogenetically independent pairs (boldface, Table 2) that show comparable levels of amino acid divergence for shared orthologs. In addition, comparisons between *E. coli* vs. *S. typhimurium* and between *B. floridanus* vs. *B. vafer* were included but are not phylogenetically independent from other pairs in the dataset.

**Table 1:** Genomes used in comparisons.

Bacterial species or strain	RefSeq ID	Abbreviation	%GC
*Blochmannia floridanus*	NC_005061	*Bloch.flor*	27.4%
*Blochmannia vafer*	NC_014909	*Bloch.vafer*	27.5%
*Blochmannia pennsylvanicus*	NC_007292	*Bloch.penn*	29.6%
*Buchnera aphidicola* str. APS *(Acyrthosiphon pisum)*	NC_002528	*Buch*APS	26.4%
*Buchnera aphidicola* str. Sg *(Schizaphis graminum)*	NC_004061	*Buch*SG	25.3%
*Escherichia coli* str. K-12 substr. MG1655	NC_000913	*E.coli*	50.8%
*Salmonella enterica* subsp. enterica serovar Typhimurium str. LT2	NC_003197	*Sal.typh*	52.2%
*Shewanella* sp. ANA-3	NC_008577	*Shew.*sp	47.9%
*Pseudomonas putida* GB-1	NC_010322	*P.putida*	61.9%
*Acinetobacter* sp. ADP1	NC_005966	*Acinet.*sp	40.1%

Genome information includes abbreviations used here and genomic GC content.

**Table 2:** Pairwise comparisons analyzed in this study.

Genome pairs
**i) ** ***Buch*** **APS - ** ***Buch*** **SG**
**ii) ** ***Bloch.flor - Bloch.penn***
iii) *Bloch.flor - Bloch.vafer*
**iv) ** ***Acinet.*** **sp - ** ***P.putida***
**v) ** ***E.coli - Shew.*** **sp.**
vi) *E.coli - Sal.typh.*

Pairs in boldface are phylogenetically independent from each other.

The Reciprocal Sequence Distance (RSD) algorithm [54] was used to identify orthologs shared within each of the six genome pairs and those shared across all ten genomes. The number of orthologs detected within each genome pair (Table 3, column 2) is based on conservative, stringent detection parameters may exclude some divergent genes. Nonsynonymous and synonymous divergences (dN and dS, respectively) were estimated using a likelihood-based approach [55] that accounts for the distinct base composition and codon structure of these species (see Methods). The median dS values illustrate that synonymous divergences far exceed saturation for all pairs, except *E. coli* vs. *S. typhimurium* where the median dS value still exceeds one (Table 3).

**Table 3:** Sequence divergences for shared orthologs detected within each genome pair.

	Pre-filter			Filtered										
genome pair	# shared orthologs within pair	med dN	med dS	# shared orthologs within pair	% genes filtered	med dN	med dS	med kappa	med *t*	med D_r_	med D_c_	med D_r_/D_c_	mean D_r_/D_c_	s.e. of D_r_/D_c_ (among genes)
i) *BuchAPS - BuchSG*	527	0.150	3.66	497	5.7%	0.153	3.73	2.22	2.09	0.155	0.229	0.700	0.714	0.0077
ii) *Bloch.flor - Bloch.penn*	578	0.214	4.32	567	1.9%	0.216	4.37	2.70	2.80	0.218	0.321	0.676	0.694	0.0076
iii) *Bloch.flor - Bloch.vafer*	571	0.185	3.27	559	2.1%	0.188	3.28	2.69	2.07	0.188	0.278	0.669	0.692	0.0087
iv) *Acinet*.sp - *P.putida*	1,812	0.487	60.34	1,809	0.2%	0.487	60.36	1.37	50	0.428	0.750	0.552	0.559	0.0025
v) *E.coli - Shew.*sp.	1,804	0.429	59.23	1,792	0.7%	0.431	59.34	1.32	50	0.380	0.666	0.570	0.577	0.0028
vi) *E.coli - Sal.typh.*	3,174	0.055	1.25	2,402	24.3%	0.071	1.40	2.09	1.32	0.059	0.118	0.510	0.528	0.0035

Values are not directly comparable among genome pairs, since they are based on different sets of genes. D_r_ and D_c_ values presented here were estimated under the classification scheme of Miyata et al. [54]. Pre-filter values (columns 2–4) include all orthologs identified within each genome pair. Filtered data (columns 5–15) excludes orthologs for which D_r_/D_c_ estimates are considered unreliable under the “high s.e. filter” (see text), typically due to zero or very few amino acid substitutions.

### D_r_/D_c_ is associated with nonsynonymous divergence

I initially applied four criteria for categorizing radical substitutions: charge, polarity, the amino acid classification by Miyata et al. [56] (noted as D_r_/D_c_-MY) that considers polarity and volume, and the classification scheme of Hanada et al. [57] (noted as D_r_/D_c_-HAN) found to correlate with K_a_/K_s_ values in mammalian genes. In order to identify which of the four classifications considered (charge, polarity, MY, and HAN) offers the most promising metric for selective constraint, I tested for an association between dN and D_r_/D_c_ under the four classifications. This follows the general approach of Hanada et al. [57], who identified a classification scheme that maximized the correlation between D_r_/D_c_ and K_a_/K_s_. In the present study, D_r_/D_c_ calculated under each classification showed a significant, positive association with dN for several genome pairs (Table 4). Of the six pairs considered, D_r_/D_c_ -polarity showed the strongest association for two pairs, D_r_/D_c_ -HAN for one pair, and D_r_/D_c_-MY for three pairs (two of which are phylogenetically independent). Based on its overall strong association with dN, D_r_/D_c_ -MY was selected for subsequent analyses.

**Table 4:** Nonparametric tests of association between dN and D_r_/D_c_, calculated under four classification schemes: charge, polarity, the scheme of Miyata et al. [56] (MY), and the scheme of Hanada et al. [57] (HAN).

		D_r_/D_c_ – Charge		D_r_/D_c_ – Polarity		D_r_/D_c_ –MY		D_r_/D_c_ -HAN	
genome pair	# orthologs used	r_s_	p	r_s_	p	r_s_	p	r_s_	p
i) *BuchAPS - BuchSG*	512	0.177	5.86E-05	**0.179**	**4.73E-05**	0.171	1.05E-04	0.097	0.029
ii) *Bloch.flor - Bloch.penn*	575	0.092	0.027	0.166	6.6E-05	**0.191**	**4.11E-06**	0.065	0.12
iii) *Bloch.flor - Bloch.vafer*	564	0.130	0.0019	0.111	8.4E-03	**0.203**	**1.16E-06**	0.065	0.12
iv) *Acinet*.sp - *P.putida*	1,810	−0.053	0.023	**0.174**	**9.0E-14**	−0.086	2.42E-04	0.060	0.01
v) *E.coli - Shew.*sp.	1,801	−0.035	0.134	0.103	1.2E-05	−0.031	1.85E-01	**0.167**	**1.11E-12**
vi) *E.coli - Sal.typh.*	2,894	0.168	9.4E-20	0.028	0.13	**0.209**	**<1.0E-25**	0.179	3.3E-22

Spearman's rho (r_s_) was used to quantify the strength and significance of the association. Boldface values indicate the classification scheme showing the strongest positive association with dN for a given genome pair. Within each pairwise comparison, proteins were filtered to remove those with zero radical and/or zero conservative amino acid changes under any of the four classification schemes for that particular pairwise comparison.

### Analysis of D_r_/D_c_-MY

Among exceptionally conserved proteins, very low values for D_r_ and/or D_c_ (such as zero or slightly above zero) made estimates of D_r_/D_c_ unreliable and resulted in high standard errors. In addition, very rarely, D_r_ or D_c_ was exceptionally high and uncorrected values exceeded 0.75, thus precluding application of the Jukes Cantor correction. The “high s.e. filter” omits orthologs with zero radical or zero conservative amino acid substitutions, orthologs for which the standard error of D_r_/D_c_ exceeded 50%, and those for which uncorrected D_r_ or D_c_ exceeded 0.75. This filter removed relatively few (<6%) of the orthologs from most pairs (Table 3). However, the filtering of 24.3% of orthologs from the *E. coli* vs. *S. typhimurium* comparison reflects the fact that many genes had low amino acid divergences and thus high standard errors of D_r_/D_c_. Regardless of classification, within each dataset D_r_ was typically less than D_c_ (illustrated for D_r_/D_c_-MY, Table 3), as expected if negative fitness consequences are more severe for radical amino acid changes than for conservative ones.

In addition to its association with dN in some genome pairs, D_r_/D_c_ -MY also shows weak though significant associations with other protein features, such as %GC, aromaticity, and GRAVY (Table S1). D_r_/D_c_ -MY shows a strong association with D_r_/D_c_ based on charge, Hanada's classification, and for some genome pairs, polarity. In *Blochmannia* and *Buchnera*, D_r_/D_c_ showed a negative association with %GC content. This may reflect the underlying association between D_r_/D_c_ and dN, since dN and %GC are negatively associated in endosymbionts. (Relatively conserved proteins retain signatures of the ancestral, moderate base composition, whereas divergent proteins show greater impacts of AT compositional biases.) An alternative explanation for the negative association between D_r_/D_c_ and %GC is that elevated AT-content inflates D_r_/D_c_ estimates; however, just the opposite is supported by simulation results below.

### Higher D_r_/D_c_ in endosymbionts suggests lower selective constraint

For direct comparisons among the genome pairs, analyses were restricted to the 276 orthologs detected across all ten genomes (Table S2). I further filtered this data to removed orthologs that did not survive the “high s.e. filter” for *any one* of the five species pairs i–v (Table 2). The resulting dataset retained a common set of 256 orthologs that span a range of metabolic functions. Application of the same filter to the *E. coli-S. typhimurium* pair retained just 119 of the 276 shared orthologs. For this genome pair, summary statistics for those 119 genes, as well as 221 genes retained by a less stringent filter, are presented. Notably, genome pairs i–v have comparable dN levels for the 256 shared genes (Table 5, column 6).

**Table 5:** Sequence divergences for orthologs shared across the ten bacterial genomes considered.

	Pre-filter			Filtered									
genome pair	# shared orthologs	med dN	med dS	# shared orthologs	med dN	med dS	med kappa	med *t*	med D_r_	med D_c_	med D_r_/D_c_	mean D_r_/D_c_	s.e. of D_r_/D_c_ (among genes)
i) *BuchAPS - BuchSG*	276	0.142	3.59	256	0.150	3.70	2.23	2.06	0.148	0.221	0.714	0.726	0.0116
ii) *Bloch.flor - Bloch.penn*	276	0.208	4.27	256	0.217	4.37	2.68	2.73	0.213	0.322	0.655	0.675	0.0099
iii) *Bloch.flor - Bloch.vafer*	276	0.184	3.18	256	0.188	3.18	2.56	2.00	0.187	0.281	0.643	0.679	0.0132
iv) *Acinet*.sp - *P.putida*	276	0.322	60.27	256	0.336	60.67	1.36	50	0.298	0.530	0.564	0.577	0.0064
v) *E.coli - Shew.*sp.	276	0.222	8.93	256	0.231	14.31	1.42	11.13	0.199	0.350	0.564	0.574	0.0079
vi) *E.coli - Sal.typh.*	276	0.021	0.62	221*	0.025	0.69	2.38	0.63	0.018	0.045	0.456	0.501	0.0196
				119	0.037	0.90	2.29	0.80	0.029	0.061	0.470	0.490	0.0160

Values are comparable among genome pairs, since they are based the same set of orthologs. D_r_ and D_c_ values were estimated under the classification scheme of Miyata et al. [56]. Pre-filter values (columns 2–4) include 276 orthologs shared among the ten genomes considered. Filtered (columns 5–15) excludes orthologs for which D_r_/D_c_ estimates are considered unreliable in *any one* of genome pairs i–v, generating a set of 256 shared orthologs. A less stringent filter was used for (vi) *E.coli-Sal.typh.*, in order to retain a comparable number of genes for comparison (see footnote).

*The 221 genes analyzed for *E. coli - S. typhimurium* included the 256 orthologs analyzed for other genome pairs, minus 35 genes that contain zero radical and/or conservative substitutions between *E. coli - S. typhimurium*, thus making D_r_/D_c_ zero or undefined. Application of the more stringent “high s.e. filter” to *E.coli - Sal.typh*. reduced this dataset to 119 genes, also summarized here.

**Figure 2:**
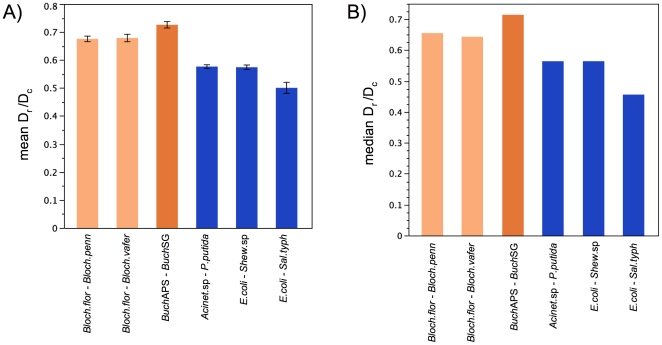
Empirical data: The two endosymbiont groups show a significant elevation in D_r_/D_c_, consistent with lower selective constraint in their small populations. Among 256 orthologs shared across all six genome pairs, (a) mean values (with bars showing standard error) and (b) median values of D_r_/D_c_ were substantially higher within pairs of endosymbionts (orange) than free-living species (blue). This elevation of D_r_/D_c_ was significant by a sign test and Wilcoxon test (see text and Table 6). D_r_/D_c_ was calculated under the classification scheme of Miyata et al. [56]. A less stringent filter was used for *E. coli*-*S. typhimurium*, in order to retain a comparable number of genes for pair. For this pair, the 221 genes analyzed consisted of the 256 orthologs surviving the “high s.e. filter” (see text) in the five other genome pairs, minus 35 genes that contain zero radical and/or conservative substitutions between *E. coli* and *S. typhimurium*.

Among the 256 shared orthologs, D_r_/D_c_ is higher for pairs of endosymbionts than for pairs of free-living gamma-Proteobacteria (Figure 2). Within *Buchnera* and within *Blochmannia*, 66–79% of genes show higher D_r_/D_c_ compared to pairs of free-living bacteria (Table 6). This elevation of D_r_/D_c_ in endosymbiont pairs is highly significant by sign and Wilcoxon tests (Table 6). While the elevation of D_r_/D_c_ in *Buchnera* compared to *Blochmannia* is significant, the difference is not nearly as extreme as that observed between endosymbiont vs. free-living bacterial pairs.

**Table 6:** Comparison of D_r_/D_c_ between pairs of bacterial species.

							(D_r_/D_c_, pair A)/(D_r_/D_c_, pair B) a			sign test				Wilcoxon test	
	pair A		pair B	# orthologs used	median D_r_/D_c_, pair A	median D_r_/D_c_, pair B	median	mean	(99% confidence interval)	D_r_/D_c_ higher in pair A	D_r_/D_c_ higher in pair B	% of genes, D_r_/D_c_ higher in pair A	p^b^	Chi Square	p^c^
**(a)**	*BuchAPS - BuchSG*	*vs.*	*E.coli - Shew*.sp.	256	0.7143	0.5643	1.29	1.32	(1.25–1.39)	196	60	76.6%	2.5E-18	101.3	8.10E-24
	*BuchAPS - BuchSG*	*vs.*	*Acinet.sp - P.putida*	256	0.7143	0.5642	1.23	1.29	(1.23–1.36)	202	54	78.9%	1.4E-21	104.4	1.70E-24
	*Bloch.flor - Bloch.penn*	*vs.*	*E.coli - Shew*.sp.	256	0.6552	0.5643	1.17	1.22	(1.17–1.28)	185	71	72.3%	3.4E-13	60.9	5.87E-15
	*Bloch.flor - Bloch.penn*	*vs.*	*Acinet.sp - P.putida*	256	0.6552	0.5642	1.17	1.21	(1.15–1.26)	189	67	73.8%	6.1E-15	61.8	3.85E-15
	*Bloch.flor - Bloch.vafer*	*vs.*	*E.coli - Shew*.sp.	256	0.6433	0.5643	1.14	1.23	(1.15–1.30)	173	83	67.6%	9.6E-09	32.4	1.23E-08
	*Bloch.flor - Bloch.vafer*	*vs.*	*Acinet.*sp - *P.putida*	256	0.6433	0.5642	1.11	1.21	(1.37–1.29)	169	87	66.0%	1.7E-07	30.6	3.25E-08
**(b)**	*E.coli - Shew*.sp.		*Acinet.*sp - *P.putida*	256	0.5643	0.5642	1.01	1.02	(0.98–1.06)	130	126	50.8%	0.803	0.2	0.697
	*BuchAPS - BuchSG*	*vs.*	*Bloch.flor - Bloch.penn*	256	0.7143	0.6552	1.08	1.12	(1.06–1.18)	147	109	57.4%	0.017	10.8	0.0010
	*BuchAPS - BuchSG*	*vs.*	*Bloch.flor - Bloch.vafer*	256	0.7143	0.6433	1.09	1.16	(1.09–1.22)	147	109	57.4%	0.017	12.2	0.00049
	*Bloch.flor - Bloch.penn*	*vs.*	*Bloch.flor - Bloch.vafer*	256	0.6552	0.6433	1.01	1.05	(1.01–1.10)	133	123	52.0%	0.532	0.7	0.388

**(a)** D_r_/D_c_ is significantly higher in endosymbionts (pair A) than in related free-living bacteria (pair B). For these comparisons, the value of [(D_r_/D_c_, pair A)/(D_r_/D_c_, pair B)] (or, the ‘elevation index’ described in the text) exceeds one, as does the lower bound of the 99% confidence interval of this index. In addition, the elevation of D_r_/D_c_ is highly significant by the sign test and Wilcoxon test.

**Figure 3:**
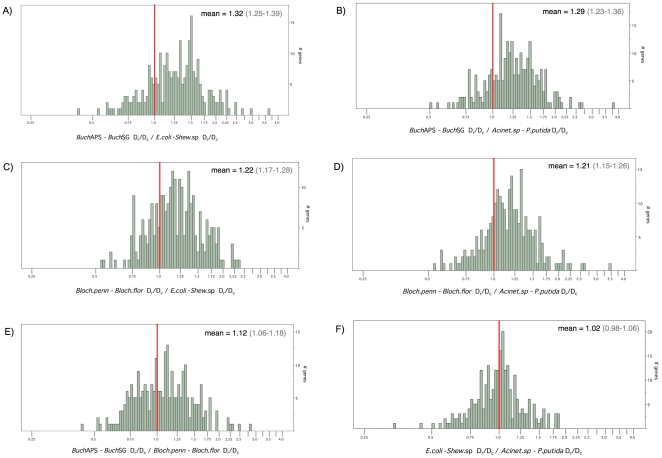
Empirical data: Frequency distributions of the ratio of two D_r_/D_c_ values illustrate elevated values in endosymbionts compared to free-living bacteria. The ratio of two D_r_/D_c_ values (or, the ‘elevation index’ described in the text) is presented along the x-axis on a log scale for ease of comparison. Mean values and 99% confidence intervals are listed for each comparison. Equality of the two D_r_/D_c_ values would give a ratio of one, indicated by the red reference line. (**a–d**) D_r_/D_c_ is substantially elevated within *Buchnera* and *Blochmannia* when either is compared to related free-living bacteria. Considered in the framework of this figure, significant sign test results for these comparisons (Table 6) indicate that significantly more genes fall above the value of one (i.e., higher D_r_/D_c_ in endosymbionts) than fall below this value. Differences were modest between (**e**) *Buchnera* and *Blochmannia* and (**f**) free-living bacterial pairs. D_r_/D_c_ was calculated under the classification scheme of Miyata et al. [56].

**Figure 4:**
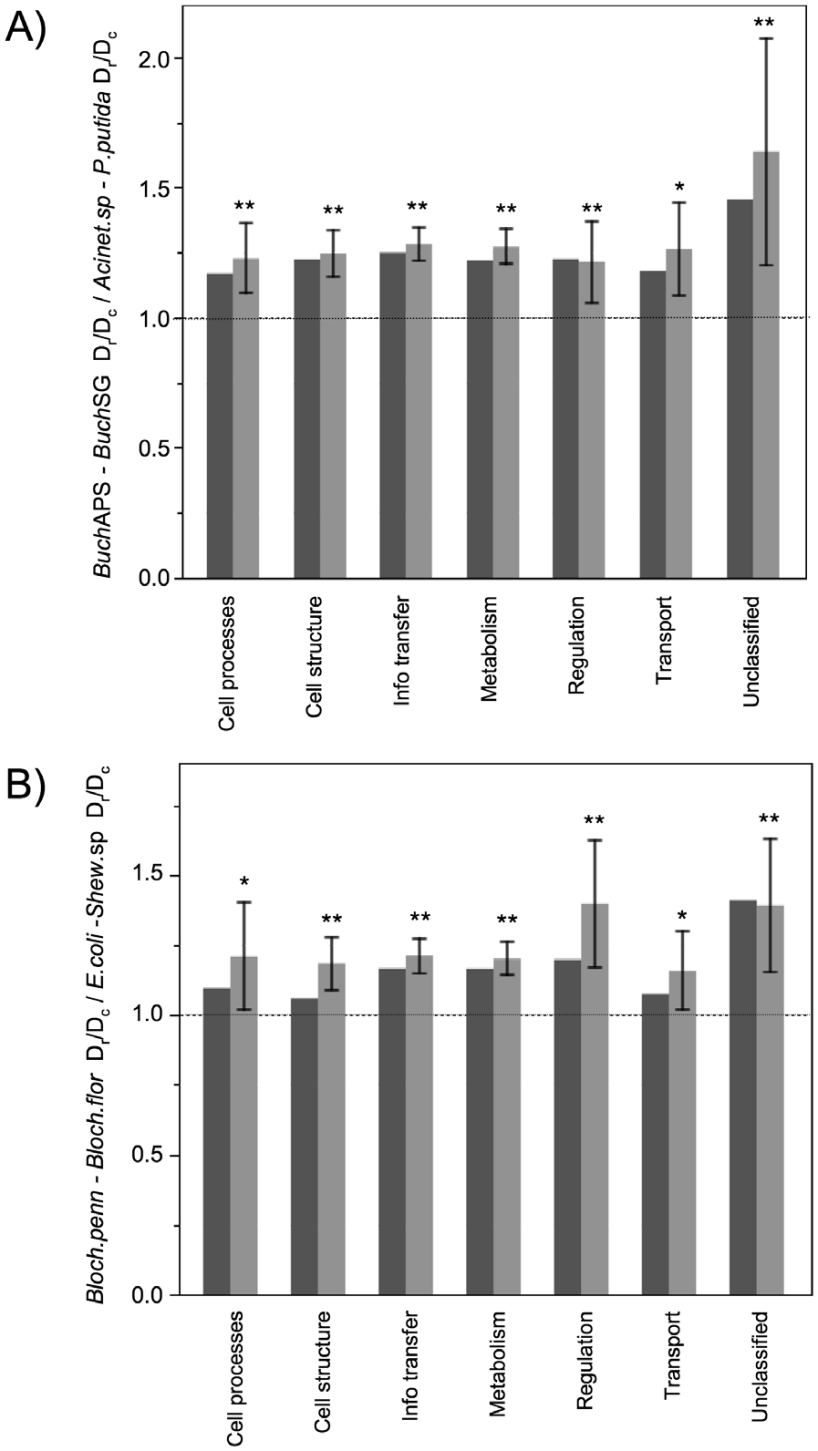
Empirical data: Elevated D_r_/D_c_ in endosymbionts is consistent across functional categories. The ratio of D_r_/D_c_ values for endosymbiont pairs divided by D_r_/D_c_ for free-living bacterial pairs (or, the ‘elevation index’ described in the text) consistently exceeds one, indicating higher D_r_/D_c_ in the endosymbionts. This pattern holds across diverse functional categories, including core cellular processes such as information transfer. Median (dark grey) and mean values (light grey, with bars showing 95% confidence intervals) are based on 256 shared orthologs shared among genomes. Asterisks indicate that the lower bound of the 95% (*) or 99% (**) confidence interval of the ‘elevation index’ exceeds one, which is marked with a hashed reference line. Functional categories are based on MultiFun assignments to *E. coli* orthologs [72], [73]. When *E. coli* genes were assigned to more than one broad category, that ortholog is represented more than once in the summary data presented. The numbers of orthologs analyzed for each functional category are as follows: Cell processes, 23; Cell structure, 73; Information transfer, 122; Metabolism, 112; Regulation, 19; Transport, 22; Unclassified, 14. Figure S1 shows the data points underlying the mean and median values presented here. Table S3 provides 99% confidence intervals and the results of sign and Wilcoxon tests, which also support significant elevation in endosymbiont D_r_/D_c_ values across functional groups.

**(b)** By contrast, differences in D_r_/D_c_ within free-living groups or within endosymbionts were more subtle. While *Buchnera* showed a higher D_r_/D_c_ ratio than *Blochmannia*, this is only marginally significant (p<0.017) by the sign test and has a relatively low ChiSquare value in the Wilcoxon test. D_r_ and D_c_values were estimated under the classification scheme of Miyata et al. [56].

a For each comparison between two genome pairs, the ratio of the two D_r_/D_c_ values is the ‘elevation index’ described in the text and represented in Figures 3 and 4. The index was calculated for *each* of the 256 orthologs and then the subsequent calculations (of median, mean, and confidence intervals) were performed.

b Significance of the sign test was evaluated using **(a)** an exact one-sided binomial test for comparisons between endosymbionts vs. free-living bacteria to test the null hypothesis that D_r_/D_c_ is not higher in the endosymbiont pair, and **(b)** using a two-way test for comparisons within endosymbionts or within free-living groups.

c Significance of the Wilcoxon test was evaluated using a ChiSquare approximation.

In order to compare D_r_/D_c_ between two genome pairs on a gene-by-gene basis, I calculated the ratio of the two D_r_/D_c_ values (one value for each pair), for each of the 256 genes examined. I refer to this ratio of D_r_/D_c_ ratios as the ‘elevation index’ for brevity. If the two genomes show no difference in D_r_/D_c_, the expected value of the elevation index is one. Figure 3 shows the distribution of this index for select comparisons, presented on a log scale for ease of visualization. The mean value and 99% confidence intervals are provided for each comparison. For the majority of genes, D_r_/D_c_ in endosymbionts is higher than the corresponding ortholog in free-living pairs (Figure 3 a–d; elevation index >1 for most genes). In each comparison of endosymbionts vs. free-living bacteria, the lower bound of the 99% confidence interval of the elevation index is substantially greater than one (Table 6; Figure 3 a–d), consistent with a substantial elevation of D_r_/D_c_ in the endosymbionts. By contrast, the index shows a more even distribution around one for *Buchnera* vs. *Blochmannia* (Figure 3e), and for *E. coli-Shewanella* sp. vs. *Acinetobacter* sp.-*P.putida* (Figure 3f), reflecting comparable D_r_/D_c_ values within these comparisons. While *Buchnera* showed a higher D_r_/D_c_ ratio than *Blochmannia*, this is only marginally significant (p<0.017) by the sign test and has a relatively low ChiSquare value in the Wilcoxon test (Table 6). In the comparison between the free-living species pairs, the 99% confidence interval of the elevation index spans one (Figure 3f) and the sign and Wilcoxon test results are nonsignificant. For all possible comparisons of two genome pairs, the above statistics are listed in Table 6.

Importantly, the 256 shared orthologs span a range of functional categories that include fundamental processes such as information transfer (DNA replication, transcription, and translation) and cell processes (cell division and cell cycle physiology). Across these various categories, endosymbiont genes show a consistent elevation in D_r_/D_c_ (Figure 4; Figure S1; Table S3). The significance of this pattern was tested in three ways. First, to determine if the elevation index (here, (D_r_/D_c_ for endosymbionts)/(D_r_/D_c_ for free-living bacteria)) was significantly greater than one, we estimated its 95% and 99% confidence intervals. The lower bound of the 95% confidence interval exceeded one for all comparisons, and the lower bound of the 99% confidence interval exceeded one for most comparisons (asterisks in Figure 3; confidence intervals listed in Table S3). In addition, for most functional categories, both the sign test and Wilcoxon test were highly significant (Table S3), indicating elevation of D_r_/D_c_ in endosymbionts. The few non-significant results were functional categories with relatively few genes and therefore small sample sizes in the statistical tests. The pervasive nature of this D_r_/D_c_ elevation in endosymbionts across diverse functional groups suggests that the underlying mechanism operates genome-wide (see Discussion).

### Sensitivity of D_r_/D_c_ estimates in simulated datasets

Potential estimation biases are critical when comparing bacteria representing different lifestyles. Namely, genomes of host-dependent species often have low %GC compared to free-living relatives (Table 1). Given that sequence composition and mutational parameters can influence D_r_/D_c_ [48], [49], I explored whether estimation biases could explain the observed elevation of D_r_/D_c_ in endosymbionts.

DNA sequence datasets were simulated under a codon model that varied in four factors. (Please see the Methods for rationale behind parameter values.) (i) Codon frequencies were especially important to consider here, as this is the factor varies among the genomes compared. Datasets were generated under equilibrium codon frequencies constrained to match the genomic codon composition of *Buchnera* APS, *Blochmannia floridanus*, or *E. coli*. This use of codon tables accounted for differences among genomes in %GC content, relative synonymous codon usage and relative amino acid usage. (ii) dN/dS was set to 0.3 or 0.6. (iii) The distance between sequences, *t*, measured as mean substitutions per site, was set to 0.5, 1, or 4. (iv) The transition/transversion ratio (kappa) was set to 1, 2, 5, or 10. All combinations of these parameters generated 72 datasets of 500 sequence pairs each. For all simulated datasets the expected D_r_/D_c_ is one, because the codon model does not distinguish radical versus conservative substitutions.

**Figure 5:**
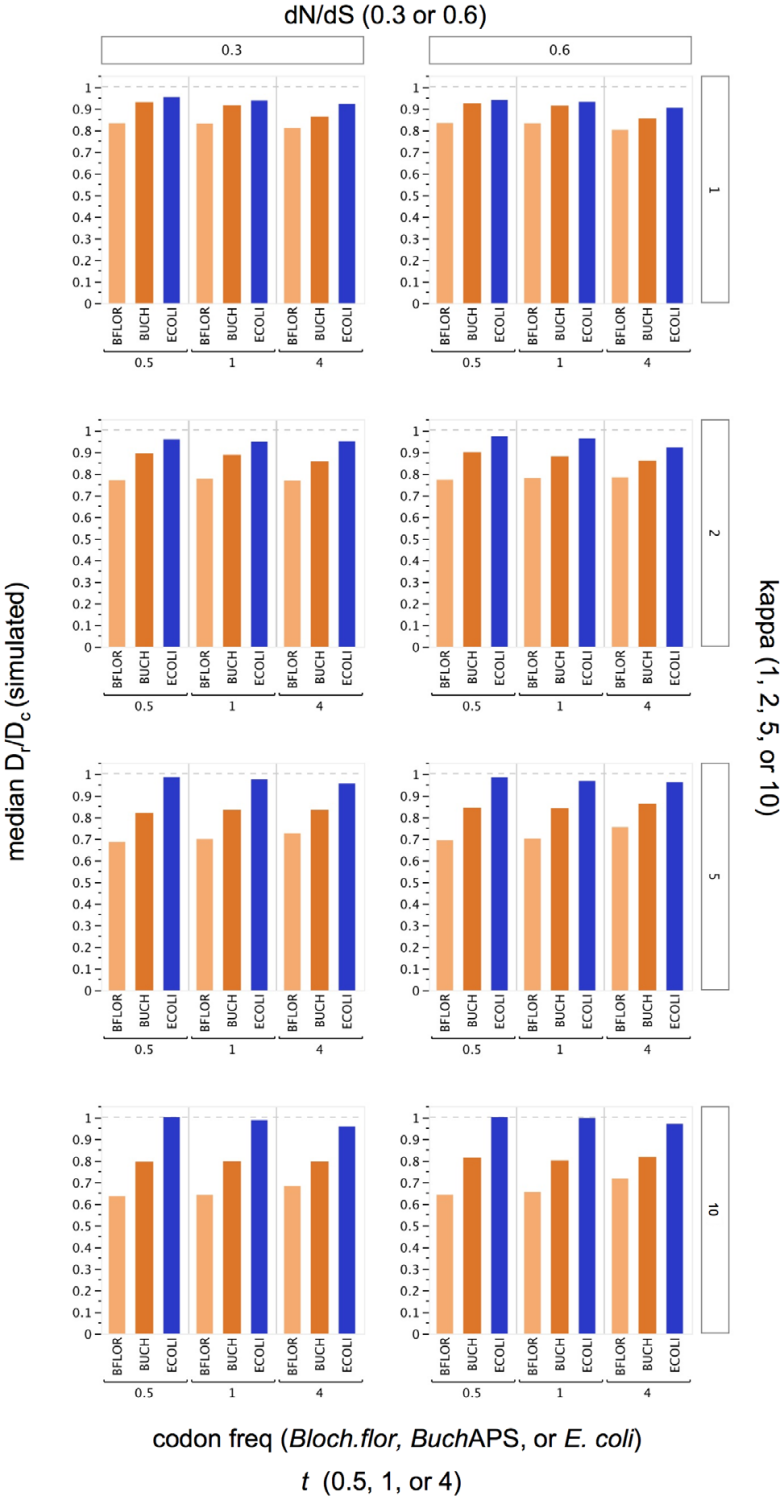
Simulated datasets show that D_r_/D_c_ estimates are sensitive to codon frequencies. The medians shown here are based on 500 sequence pairs for each of the 72 simulated datasets. The expected D_r_/D_c_ is one (marked with hashed line) because the codon model used in simulation did not distinguish radical versus conservative substitutions. Four parameters were varied in the simulation: codon frequencies (constrained to match those of *Buchnera* APS, *Blochmannia floridanus*, or *E. coli* codon tables), the dN/dS ratio, the distance between sequences (*t*), and the transition/transversion ratio (kappa). D_r_/D_c_ estimates were substantially suppressed in datasets simulated under endosymbiont codon frequencies, compared to datasets simulated under *E. coli* codon frequencies.

**Figure 6:**
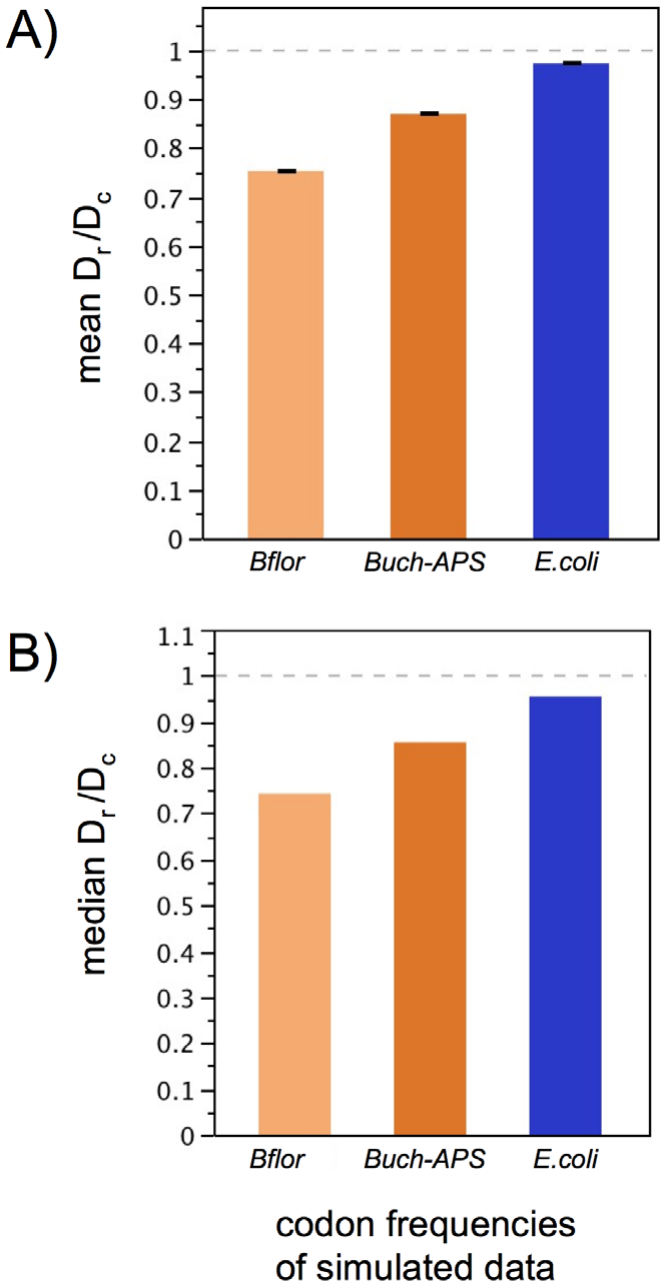
In simulated datasets, use of endosymbiont codon frequencies suppress D_r_/D_c_ estimates. The chart represents a simplified version of Figure 5, pooling across other simulation parameters (kappa, *t*, and dN/dS) to illustrate the significant effect of codon frequencies. (**a**) Mean values (with bars indicating standard errors) and (**b**) median values of D_r_/D_c_ are suppressed in datasets simulated under endosymbiont codon frequencies. Notably, these estimation biases occur in the opposite direction as the patterns observed in empirical genome comparisons, thereby making the inference elevated D_r_/D_c_ in endosymbionts more conservative.

Visual inspection of the median D_r_/D_c_ values shows that many values deviate from one, reflecting biases in the estimate (Figure 5). Codon frequencies have a consistent and large effect (Figure 6). In particular, sequences simulated under the AT-rich codon frequencies of endosymbionts showed a considerable reduction in D_r_/D_c_ estimates. This bias was exacerbated as kappa increased. Varying *t* or dN/dS had less of an effect. To quantify the sensitivity of D_r_/D_c_ estimates to the four simulation parameters, I used a multi-way analysis of variance (ANOVA). F values (Table 7) reflect the magnitude of the effects and illustrate a high sensitivity of D_r_/D_c_ to codon frequencies (F value of 5,323). ANOVA also points to secondary but highly significant effects of kappa and *t*.

**Table 7:** Results of multiway ANOVA testing the sensitivity of D_r_/D_c_ estimates to the four simulation parameters.

	df	Sum of Squares	F	p
codon frequencies	2	292.749	5312.15	0*
kappa	3	23.205	280.72	3.95E-180
*t*	2	4.620	83.84	4.70E-37
dN/dS	1	0.003	0.09	0.76

*p-value fell below the lowest threshold of the software used. Supporting Information Legends.

Under most circumstances, the high sensitivity of D_r_/D_c_ estimates to codon frequencies would caution against comparing D_r_/D_c_ across sequences with compositional differences. However, in this particular study, the trend observed in simulations is *just the opposite* of patterns observed in genome comparisons. That is, sequences simulated under AT-rich endosymbiont codon frequencies show a significant deflation of D_r_/D_c_ estimates, whereas empirical datasets point to elevated D_r_/D_c_ in *Blochmannia* and *Buchnera*. In this sense, estimation biases make the conclusion of higher D_r_/D_c_ in endosymbionts more conservative.

## Discussion

Numerous empirical studies support the prediction of the nearly neutral model: the efficiency with which selection can remove harmful variants depends on N_e._ Across vertebrates, invertebrate, plants, fungi, and bacteria, a reduction in N_e_ is often coupled with reduced selective constraint and accelerated accumulation of deleterious changes. These inferences rely on measures of selective constraint, most commonly K_a_/K_s_ or related indices. However, K_a_/K_s_ is unreliable across deep sequence divergences where synonymous substitution rates are saturated.

Here, I explored the utility of D_r_/D_c_, an alternative index for selective constraint that is based on physiochemical effects of amino acid changes. D_r_/D_c_ offers a tool to compare selective constraint across species that are highly divergent and therefore not amenable to accurate estimates of synonymous divergences. D_r_/D_c_ may be especially useful for genome sequence comparisons, since genomes are often selected to span broad phylogenetic and metabolic diversity rather than to facilitate comparisons among closely-related taxa. Although D_r_ and D_c_ (and their ratio) are prone to estimation biases, earlier work shows that D_r_/D_c_ behaves as expected for an index of selective constraint. For example, measures of D_r_/D_c_ show a positive association with K_a_/K_s_ [52], [57], suggesting that proteins under relaxed selective constraint accumulate more nonsynonymous changes and, of these changes, a greater proportion are radical. Earlier work also shows that an increase in radical substitutions corresponds to other signatures of reduced selective constraint, such as a higher rate of indels and increased proportion of variable sites [58]. Moreover, several studies have documented higher rates of radical substitutions in primates than in rodents, suggesting that purifying selection against radical changes is less effective in relatively small primate populations [4], [16], [48], [52].

The present study shows that D_r_/D_c_ behaves similarly in bacterial species and therefore offers a promising gauge for selective constraint. First, within genome pairs, D_r_/D_c_ shows a modest but significant, positive association with rates of protein evolution (measured here as dN) in most genome pairs considered here. Although exceptions exist, the overall trend suggests that proteins under relaxed functional constraint experience a greater proportion of radical substitutions.

Second, D_r_/D_c_ is higher in the obligate endosymbionts *Blochmannia* and *Buchnera*, compared to orthologous genes in related free-living bacteria. This elevation of D_r_/D_c_ was highly significant by Wilcoxon and sign tests. Data simulations highlight biases in the estimation of D_r_/D_c_; however, these biases do not explain the primary result of elevated D_r_/D_c_ in endosymbionts. That is, simulated datasets constrained to match the AT-rich, endosymbiont codon frequencies show lower, rather than higher, D_r_/D_c_ values. In light of the simulation results, it is likely that D_r_/D_c_ in endosymbionts is even higher than estimated here.

The observed elevation of D_r_/D_c_ in the two endosymbiont groups is consistent with other genomic features pointing to the fixation of slightly deleterious mutations in these lineages. Such features include: rapid rates of DNA sequence and protein evolution, a concentration of this rate increase at nonsynonymous sites, and reduced structural stability of proteins and of ribosomal RNA [17], [44], [59]. Furthermore, population genetic studies point to reduced intraspecific variation, an excess of nonsynonymous polymorphisms, and non-neutral distributions of allele frequencies all consistent with deleterious evolution in small populations [41], [42]. These results from population-level studies are not predicted nor explained by the alternative hypothesis of relaxed purifying selection; the results are, however, expected consequences of genetic drift in small populations.

Combined with these other genome features, increased D_r_/D_c_ in *Buchnera* and *Blochmannia* further supports the hypothesis that obligate endosymbionts are vulnerable to stochastic effects of drift due to small N_e_. Mutation pressure alone cannot account for the observed elevation in the relative frequency of radical amino acid substitutions, as simulation results indicate that strong AT compositional bias, in and of itself, does not elevate D_r_/D_c_. While relaxed purifying selection in endosymbionts may contribute to higher D_r_/D_c_, shifts in selection coefficients are typically expected to affect specific genes or functional groups and are unlikely to fully explain a pervasive elevation in D_r_/D_c_ across multiple functional categories. Although alternative processes may play contributing roles as discussed in more depth below, I view elevated D_r_/D_c_ as best explained by a genome-level process such as genetic drift in small endosymbiont populations.

### Alternative explanations for elevated D_r_/D_c_

Additional processes may contribute to the distinct genomic features of endosymbionts that are often attributed to genetic drift (e.g., rapid evolutionary rates, elevated K_a_/K_s_, and elevated D_r_/D_c_ shown here). First, changes in population size often correspond to shifts in ecological niche, and consequently, changes in selection coefficients. Any study testing the effects of N_e_ differences in natural populations, including studies of mammals, insects and bacteria, faces the limitation of potential coincident shifts in selective pressures. This is certainly true in comparisons of endosymbiotic vs. free-living bacteria. Endosymbiotic bacteria may experience relaxed purifying selection, perhaps due to the relatively stable intracellular environment they occupy. Relaxed selection on gene function played a role in early gene loss and metabolic streamlining of small endosymbiont genomes [60]. Among genes that are retained, relaxed purifying selection on particular functional categories may contribute to the observed elevation in evolutionary rates, K_a_/K_s_, and D_r_/D_c_.

This said, relaxed purifying selection is unsatisfying as a sole explanation for the pervasive, genome-wide patterns in endosymbionts. Importantly, relaxed selection is expected to affect specific genes or functional groups, rather than have a genome-wide effect across multiple functional categories. Following this logic, previous studies have argued that the pervasive rate acceleration and elevated K_a_/K_s_ are unlikely due to relaxed selection alone, but rather reflect the genome-wide process of genetic drift in small populations [45]–[47]. Similarly, genome-wide mechanisms such as genetic drift best explain the consistent elevation in D_r_/D_c_ observed here. The 256 orthologs analyzed here were selected because they are shared across the ten genomes considered. As expected for persistent genes, encoded functions include core processes such as information transfer (transcription, translation and replication) and cell processes (which includes cell division and cell cycle physiology), along with a range of other functional categories. One might imagine relaxed selection on particular metabolic functions with limited importance in host cellular environment. For example, relaxed selection may contribute to the more severe elevation in D_r_/D_c_ among the small number (fourteen) of unclassified orthologs included (Figure 4). However, it is more difficult to imagine that an endosymbiotic lifestyle reduces selection coefficients across core cellular processes.

As a second alternative explanation of elevated D_r_/D_c_ in endosymbionts, constitutive overexpression of the chaperonin GroEL [61], might offer compensatory mechanism to cope with deleterious substitutions and therefore facilitate the accumulation of such changes in endosymbiont genomes. That is, GroEL and other molecular chaperones may act as buffers (i.e., capacitors) for evolution by allowing substrate proteins to accumulate mutations but still fold correctly [17], [62], [63]. A recent study indicates an important role of chaperonins on the evolution of interacting proteins [64], although other work found no evidence that GroEL acts as a capacitor for evolutionary change [65]. While intriguing, the potential role of GroEL as a capacitor in endosymbiont evolution remains speculative [65].

Third, it is possible that linkage to positively selected sites may contribute to elevated D_r_/D_c_ and other genome features of endosymbionts. Because *Buchnera* and *Blochmannia* are strictly asexual, the entire genome is linked. In this situation, linkage to selected sites may have profound and pervasive consequences. For instance, the elimination of strongly deleterious mutations may reduce variation elsewhere in the genome and contribute to the accumulation of slightly deleterious mutations. In addition, growing evidence suggests that positive selection influences many endosymbiont proteins [66], [67]. Due to linkage, positive selection anywhere in the genome would purge variation elsewhere and any slightly deleterious changes would become fixed along with the beneficial mutation. This effect of *genetic draft* [33] is potentially significant in endosymbionts but largely unexplored, perhaps because this force is difficult to distinguish from genetic drift in small populations.

### Conclusions

In sum, this study shows that D_r_/D_c_ offers a promising gauge of selective constraint in bacteria. Specifically, the ratio shows the predicted elevation in species that are prone to the accumulation of deleterious changes. Previous work has shown that endosymbionts show a pervasive, genome-wide rate acceleration and elevated K_a_/K_s_. Likewise, we found that D_r_/D_c_ is consistently elevated in endosymbionts and affects numerous functional categories, including fundamental cellular processes. Relaxed purifying selection on particular functions may contribute to the observed elevation in D_r_/D_c_, but relaxed selection alone is not satisfying as a sole explanation for this pervasive phenomenon. Rather, the results are more consistent with genome-wide processes such as genetic drift in small populations.

The taxonomic scope of the present study was limited by the to need to simulate datasets that mimic the codon frequencies as the genomes compared here. These simulations eliminate the possibility that estimation biases explain the observed elevation of D_r_/D_c_ in endosymbionts. However, the observation of biases highlights important challenges for the broader use of D_r_/D_c_ across genomes, particularly those that vary in sequence composition. In future work, the development of new methods to estimate D_r_/D_c_ more accurately will broaden the utility of this index, in the same way that gradual improvements to K_a_ and K_s_ estimates have made K_a_/K_s_ a broadly useful metric.

## Methods

### Genome comparisons

#### Ortholog identification

Chromosomal sequences and annotations were downloaded from NCBI Genbank on February 1, 2010–January 1, 2011 for the ten genomes listed in Table 1. The Reciprocal Sequence Distance (RSD) algorithm [54] was used to identify the reciprocal best BLAST hits (rbh) between translated ORFs. This program uses blastp to identify potential matches of a given translated gene, aligns all potential matches using ClustalW [68], and calculates a maximum likelihood estimation of amino acid substitutions between proteins by invoking PAML 4.2 [55]. Protein divergences were based on an empirical amino acid substitution rate matrix [69] and accounted for variation in evolutionary rates among protein sites using a gamma distribution with shape parameter alpha = 1.53 (as recommended by Smith [69]). The protein with the lowest divergence was then compared to the first genome using blastp, followed by the alignment and divergence calculations. If the protein match with the lowest divergence was the same as the original query sequence, the pair was considered orthologous and the divergence was retained in the output. I used stringent criteria in RSD of retaining only blastp matches of E<10^−10^ (with the RSD parameter thresh = 1e-10), and requiring that the alignable region of two sequences exceed 80% of the alignment's total length (RSD parameter div = 0.8). These stringent criteria may generate false negatives, by excluding true orthologs that are highly divergent. Such comparisons were performed within each of the pairs listed in Table 2. All genomes were then compared to *E. coli* using RSD as outlined above, in order to identify orthologs shared across the ten genomes.

#### Molecular evolutionary analysis

Within each genome pair, shared orthologs were aligned using transalign.pl [70], a script that invokes ClustalW [68] to perform a amino-acid based alignment and back-translates to the nucleotide sequence. Each pair of aligned nucleotide sequences was analyzed in the codeml package of PAML 4.2 [55] using pairwise analysis (runmode = −2) to obtain ML estimations of dS and dN and their standard errors, the transition/transversion ratio (kappa), and the total distance (*t*) between sequences. Estimates of dS and dN accounted for the distinct base compositions and codon structures of the genomes considered. Specifically, the equilibrium frequencies of codons were calculated from the nucleotide frequencies at the three codon positions (CodonFreq = 2). CodonW (http://codonw.sourceforge.net/) was used to quantify the %GC content, aromaticity, and GRAVY for each gene.

The per-site rate of radical and conservative substitutions (D_r_ and D_c_) and their standard errors were estimated with the Hon-New program, which applies the method of Zhang [52] to account for the transition/transversion bias. D_r_ and D_c_ was calculated based on four classification schemes: charge, polarity, the amino acid classification by Miyata et al. [56] that considers polarity and volume, and the classification scheme of Hanada et al. [57]. Amino acid groups under each classification scheme are shown in Table S4. For each gene pair, information about kappa (as estimated above in codeml) was considered in the D_r_ and D_c_ estimations. D_r_ and D_c_ values were adjusted with a Jukes Cantor correction [71], using the formula D_corr_ = −0.75*LN(1−(4/3*D_orig_)), where D_orig_ is the uncorrected divergence value and D_corr_ is the corrected value.

#### Data filter

Under the circumstances listed below, I considered D_r_/D_c_ estimates as unreliable and excluded them under the “high s.e. filter.” First, this filter excludes particularly conserved genes that had zero radical and/or conservative amino acid changes, or so few amino acid substitutions that the percent standard error of D_r_/D_c_ exceeded 50% of the D_r_/D_c_ ratio itself. In addition, the Jukes Cantor correction cannot be applied if D_orig_>0.75 since the natural log of zero or a negative number is undefined. The very few genes with such high divergence were also excluded as part of the “high s.e.” filter.

The ortholog detection methods detailed above identified 276 orthologs shared across the ten genomes considered. Twenty of those orthologs were excluded because they did not pass the “high s.e. filter” in one or more of the genome pairs i–iv (Table 2). Subsequent comparisons among bacterial genome pairs focused primarily upon the remaining 256 shared orthologs that survived the filter in each of the five pairs. The recently-diverged *E. coli* vs. *S. typhimurium* pair had numerous genes with very low D_r_ and/or D_c_ values for which the standard error of D_r_/D_c_ exceeded 50%. This pair was filtered less stringently in order to retain a comparable number of genes (see text and Table 5 legend).

#### Assignment of functional categories

The 256 shared orthologs were assigned broad functional categories based on the MultiFun assignment of *E. coli* orthologs [72], [73], downloaded from http://genprotec.mbl.edu/. Multifun provides detailed classification system for the physiological and cellular roles of gene products. For the purpose of this broad analysis, I considered only the highest level category, represented by the first number in the Multifun assignment, and I did not consider cellular location (represented under Multifun category 7). Broad functional classes considered here included: Cell processes, Cell structure, Information transfer, Metabolism, Regulation, Transport, and Unclassified (no Multifun assignment other than Location in some instances). Several *E. coli* genes are assigned to more than one category. The specific functions within each broad category are listed on the Multifun website, at http://genprotec.mbl.edu/files/MultiFun.txt.

### Data simulations

I used simulations to explore the sensitivity of D_r_/D_c_ estimates to differences in sequence composition and patterns of nucleotide change. Following the general approach of Smith [48] and Dagan [49], I simulated sequence datasets that varied in their equilibrium codon composition frequencies and various mutational parameters. These datasets were generated with the evolver program of PAML using a codon model of substitution. Since the simulations do not distinguish between radical versus conservative amino acid substitutions, the expected D_r_/D_c_ for these datasets is one, and deviations from one suggest a bias in the estimate.

In more detail, the following four factors were varied in the simulation. (i) Datasets were generated under three equilibrium codon frequencies. Three codon tables were developed from codon frequencies of all coding regions of the *Buchnera* APS, *Blochmannia floridanus*, or *E. coli* genomes. Raw codon usage number for all loci were calculated in codonW, and the number of each codon was converted to its relative frequency among the 61 possible codons. (Stop codons are excluded in this analysis.) This representation of codon usage accounts for differences in %GC, amino acid frequencies, and relative synonymous codon usage. (ii) The dN/dS ratio was set to 0.3 or 0.6, the values also selected by Smith [48]. The lower value (0.3) falls within the range of empirical K_a_/K_s_ estimates for endosymbionts genes [47]. (iii) The distance between sequences, *t*, measured as mean substitutions per site, was set to 0.5, 1, or 4, and (iv) the transition/transversion ratio (kappa) was set to 1, 2, 5, or the exceptionally high value of 10. For both *t* and kappa, the lower parameter values span the medians estimated from the empirical datasets used (Tables 3 and 5). The higher parameter values allowed us to test the effect of more extreme values on simulation results.

The combinations of all parameter settings resulted in 72 total datasets (3×2×3×4). Each simulated dataset contained 500 pairs of sequences that were 1,200 nucleotides long. In total, 36,000 sequence pairs were generated. Each sequence pair was analyzed using codeml and Hon-New, as described above for empirical genome comparisons. The sensitivity of D_r_/D_c_-MY estimates to the four simulation parameters was tested with a multi-way analysis of variance (ANOVA).

### Statistical analyses

Data tables from genome comparisons and simulations were concatenated and imported into JMP 8.0.1 (SAS Institute) for summary calculations and statistical analyses such as nonparametric tests of association, the sign test, and ANOVA.

## Supporting Information

Figure S1 **Elevated D_r_/D_c_ in endosymbionts is consistent across functional categories (individual data points).** Data points underlie the median and mean values presented in Figure 4. The y-axis (on log scale) shows the ratio of D_r_/D_c_ values for endosymbiont pairs and free-living bacterial pairs, for individual genes. This value typically exceeds one, indicating higher D_r_/D_c_ in the endosymbionts across diverse functional categories that include core cellular processes. Data include the 256 shared orthologs shared among the genomes considered. Functional categories are based MultiFun classification of the *E. coli* ortholog [72], [73]. When a given gene is assigned to more than one broad category, it is represented more than once in the data points shown here. The numbers of orthologs within each functional category are listed in the legend of Figure 4.
(TIF)

Table S1 **Nonparametric tests of association between D_r_/D_c_ and various sequence features.**
(PDF)

Table S2 **Gene IDs for the orthologs shared among the genomes considered.**
(PDF)

Table S3 **Comparison of D_r_/D_c_ between endosymbiotic vs. free-living bacterial species, across seven functional categories.**
(PDF)

Table S4 **Amino acid classification schemes used in this study.**
(PDF)
