# Supplementary figures and images for: Reduced Selective Constraint in Endosymbionts: Elevation in Radical Amino Acid Replacements Occurs Genome-Wide
Source: PLoS One. 2011 Dec 14;6(12):e28905. doi: 10.1371/journal.pone.0028905 (PMC3237559; doi:10.1371/journal.pone.0028905)

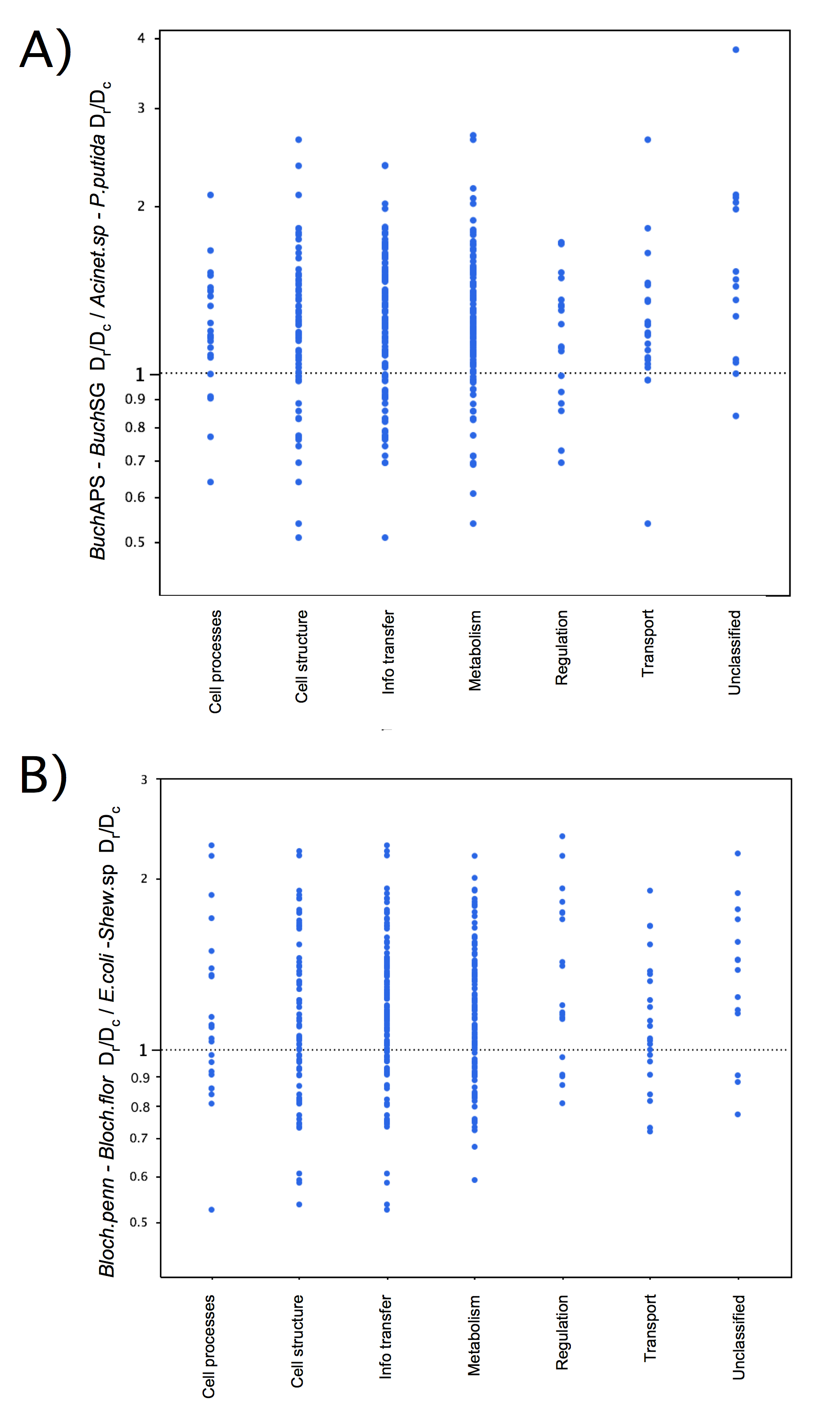

Supplement: Figure S1 — Elevated Dr/Dc in endosymbionts is consistent across functional categories (individual data points). Data points underlie the median and mean values presented in Figure 4. The y-axis (on log scale) shows the ratio of Dr/Dc values for endosymbiont pairs and free-living bacterial pairs, for individual genes. This value typically exceeds one, indicating higher Dr/Dc in the endosymbionts across diverse functional categories that include core cellular processes. Data include the 256 shared orthologs shared among the genomes considered. Functional categories are based MultiFun classification of the E. coli ortholog [72], [73]. When a given gene is assigned to more than one broad category, it is represented more than once in the data points shown here. The numbers of orthologs within each functional category are listed in the legend of Figure 4. (TIF) [file pone.0028905.s001.tif]
