# Supplementary material for: Reduced Selective Constraint in Endosymbionts: Elevation in Radical Amino Acid Replacements Occurs Genome-Wide
Source: PLoS One. 2011 Dec 14;6(12):e28905. doi: 10.1371/journal.pone.0028905 (PMC3237559; doi:10.1371/journal.pone.0028905)
Supplement: Table S1 — Nonparametric tests of association between Dr/Dc and various sequence features. (PDF) [file pone.0028905.s002.pdf]

Supplementary Table S1. Nonparametric tests of association between D_r/D_c-MY and various sequence features. See Table 3 legend for description of orthologs included. Spearman's rho (r_s) was used to quantify the strength and significance of the association. Associations significant at the p<0.005 level are marked in bold.

		dN		%GC of gene		aromaticity		GRAVY		D_r/D_c-Charge		D_r/D_c-Polarity		D_r/D_c-HAN	
genome pair	# orthologs used	r_s	p	r_s	p	r_s	p	r_s	p	r_s	p	r_s	p	r_s	p
*Buch*APS - *Buch*SG	512	**0.171**	**0.0001**	**-0.206**	**2.6E-06**	0.018	0.689	-0.016	0.714	**0.412**	**2.0E-22**	-0.006	0.889	**-0.151**	**0.001**
Bloch.flor - Bloch.penn	575	**0.191**	**4.1E-06**	**-0.196**	**2.2E-06**	0.055	0.186	**-0.125**	**0.003**	**0.486**	**<1.0E-25**	**0.135**	**0.001**	**-0.134**	**0.001**
*Acinet.*sp - *P.putida*	1,810	**-0.087**	**0.0002**	0.052	0.028	**-0.207**	**4.9E-19**	**-0.077**	**0.001**	**0.411**	**<1.0E-25**	**0.077**	**0.001**	**-0.182**	**<1.0E-25**
*E.coli - Shew.*sp.	1,801	-0.031	0.185	**0.084**	**0.0004**	**-0.117**	**6.5E-07**	-0.064	0.006	**0.430**	**<1.0E-25**	**0.097**	**3.9E-05**	**-0.145**	**7.0E-10**
E.coli - Sal.typh.	2,894	0.209	**<1.0E-25**	-0.014	0.453	**-0.089**	**1.8E-06**	**-0.083**	**8.9E-06**	**0.513**	**<1.0E-25**	0.034	0.067	**-0.137**	**<1.0E-25**
