# Supplementary material for: Reduced Selective Constraint in Endosymbionts: Elevation in Radical Amino Acid Replacements Occurs Genome-Wide
Source: PLoS One. 2011 Dec 14;6(12):e28905. doi: 10.1371/journal.pone.0028905 (PMC3237559; doi:10.1371/journal.pone.0028905)
Supplement: Table S2 — Gene IDs for the orthologs shared among the genomes considered. (PDF) [file pone.0028905.s003.pdf]

Supplementary Table S2. Gene IDs for the orthologs shared among the genomes considered. The 276 genes listed were detected in each of the ten genomes considered. Among these genes, twenty were filtered because they did not survive the "high s.e. filter" (see text) in one or more of the genome pairs i-v (Table 1b). These twenty filtered genes are shaded in grey across all columns, and the remaining 256 orthologs were the basis for most analyses. In addition, within the relatively close *E. coli- S. typhimurium* comparison, several additional genes did not survive the filter an are shaded in grey in the *S. typhimurium* ("*Sal.typh*") ID column. A conservative treatment of the *E. coli- S. typhimurium* dataset excludes such proteins, leaving 119 proteins for this pair.

E.coli bnum	*E.coli* gene name	*E. coli* COG	*Bflor* ID	*Bpenn* ID	*Bvafer* ID	*Buch*APS ID	*Buch*SG ID	*P.putida* ID	*Acinet*.sp ID	*Shew*.sp ID	*Sal.typh* ID	*E. coli* protein description
b0014	*dnaK*	COG0443O	Bfl114_dnaK	BPEN_118_dnaK	BVAF_115_dnaK	BUsg146_dnaK	BU153_dnaK	PputGB1_4728_dnaK	ACIAD3654_dnaK	Shewana3_0959_dnaK	STM0012_dnaK	chaperone Hsp70, co-chaperone with DnaJ
b0015	*dnaJ*	COG0484O	Bfl115_dnaJ	BPEN_119_dnaJ	BVAF_116_dnaJ	BUsg145_dnaJ	BU152_dnaJ	PputGB1_4727_-	ACIAD3621_dnaJ	Shewana3_0960_-	STM0013_dnaJ	chaperone Hsp40, co-chaperone with DnaK
b0023	*rpsT*	COG0268J	Bfl116_rpsT	BPEN_120_rpsT	BVAF_117_rpsT	BUsg144_rpsT	BU151_rpsT	PputGB1_0646_rpsT	ACIAD1389_rpsT	Shewana3_3140_rpsT	STM0043_rpsT	30S ribosomal subunit protein S20
b0025	*ribF*	COG0196H	Bfl117_ribF	BPEN_121_ribF	BVAF_118_ribF	BUsg143_ribF	BU150_ribF	PputGB1_0648_-	ACIAD0023_ribF	Shewana3_3137_-	STM0045_ribF	bifunctional riboflavin kinase/FAD synthetase
b0026	*ileS*	COG0060J	Bfl118_ileS	BPEN_122_ileS	BVAF_119_ileS	BUsg142_ileS	BU149_ileS	PputGB1_0649_ileS	ACIAD0022_ileS	Shewana3_3136_ileS	STM0046_ileS	isoleucyl-tRNA synthetase
b0027	*lspA*	COG0597MU	Bfl119_lspA	BPEN_123_lspA	BVAF_120_lspA	BUsg141_lspA	BU148_lspA	PputGB1_0650_lspA	ACIAD0021_lspA	Shewana3_3135_lspA	STM0047_lspA	prolipoprotein signal peptidase (signal peptidase II)
b0031	*dapB*	COG0289E	Bfl121_dapB	BPEN_125_dapB	BVAF_122_dapB	BUsg139_dapB	BU146_dapB	PputGB1_4726_-	ACIAD3619_dapB	Shewana3_0966_-	STM0064_dapB	dihydrodipicolinate reductase
b0032	*carA*	COG0505EF	Bfl122_carA	BPEN_126_carA	BVAF_123_carA	BUsg138_carA	BU145_carA	PputGB1_4725_-	ACIAD2860_carA	Shewana3_0967_-	STM0066_carA	carbamoyl phosphate synthetase small subunit, glutamine amidotransferase
b0033	*carB*	COG0458EF	Bfl123_carB	BPEN_127_carB	BVAF_124_carB	BUsg137_carB	BU144_carB	PputGB1_4724_carB	ACIAD2861_carB	Shewana3_0968_carB	STM0067_carB	carbamoyl-phosphate synthase large subunit
b0048	*folA*	COG0262H	Bfl124_folA	BPEN_128_folA	BVAF_125_folA	BUsg136_folA	BU143_folA	PputGB1_5184_-	ACIAD0514_folA	Shewana3_3214_-	STM0087_folA	dihydrofolate reductase
b0049	*apaH*	COG0639T	Bfl125_apaH	BPEN_129_apaH	BVAF_126_apaH	BUsg135_apaH	BU142_apaH	PputGB1_0430_apaH	ACIAD3008_apaH	Shewana3_3211_apaH	STM0088_apaH	diadenosine tetraphosphatase
b0051	*rsmA*	COG0030J	Bfl126_ksgA	BPEN_130_ksgA	BVAF_127_ksgA	BUsg134_ksgA	BU141_ksgA	PputGB1_0432_ksgA	ACIAD3009_ksgA	Shewana3_3209_ksgA	STM0090_ksgA	S-adenosylmethionine-6-N',N'-adenosyl (rRNA) dimethyltransferase
b0082	*mraW*	COG0275M	Bfl134_yabC	BPEN_138_mraW	BVAF_135_mraW	BUsg218_yabC	BU224_yabC	PputGB1_4520_mraW	ACIAD3368_mraW	Shewana3_3751_mraW	STM0120_mraW	S-adenosyl-dependent methyltransferase activity on membrane-located substrates
b0084	*ftsI*	COG0768M	Bfl136_ftsI	BPEN_140_ftsI	BVAF_137_ftsI	BUsg216_ftsI	BU222_ftsI	PputGB1_4518_-	ACIAD3366_ftsI	Shewana3_3749_-	STM0122_ftsI	transpeptidase involved in septal peptidoglycan synthesis (penicillin-binding protein 3)
b0088	*murD*	COG0771M	Bfl140_murD	BPEN_144_murD	BVAF_141_murD	BUsg212_murD	BU218_murD	PputGB1_4514_murD	ACIAD0270_murD	Shewana3_3745_murD	STM0126_murD	UDP-N-acetylmuramoyl-L-alanine:D-glutamate ligase
b0089	*ftsW*	COG0772D	Bfl141_ftsW	BPEN_145_ftsW	BVAF_142_ftsW	BUsg211_ftsW	BU217_ftsW	PputGB1_4513_-	ACIAD0271_ftsW	Shewana3_3744_-	STM0127_ftsW	integral membrane protein involved in stabilizing FstZ ring during cell division
b0090	*murG*	COG0707M	Bfl142_murG	BPEN_146_murG	BVAF_143_murG	BUsg210_murG	BU216_murG	PputGB1_4512_murG	ACIAD3517_murG	Shewana3_3743_murG	STM0128_murG	N-acetylglucosaminyl transferase
b0094	*ftsA*	COG0849D	Bfl145_ftsA	BPEN_149_ftsA	BVAF_146_ftsA	BUsg207_ftsA	BU213_ftsA	PputGB1_4508_-	ACIAD3512_ftsA	Shewana3_3740_-	STM0132_ftsA	ATP-binding cell division protein involved in recruitment of FtsK to Z ring
b0095	*ftsZ*	COG0206D	Bfl146_ftsZ	BPEN_150_ftsZ	BVAF_147_ftsZ	BUsg206_ftsZ	BU212_ftsZ	PputGB1_4507_-	ACIAD3511_ftsZ	Shewana3_3739_-	STM0133_ftsZ	GTP-binding tubulin-like cell division protein
b0098	*secA*	COG0653U	Bfl148_secA	BPEN_152_secA	BVAF_149_secA	BUsg195_secA	BU201_secA	PputGB1_4504_secA	ACIAD0648_secA	Shewana3_3735_-	STM0136_secA	preprotein translocase subunit, ATPase
b0114	*aceE*	COG2609C	Bfl153_aceE	BPEN_158_aceE	BVAF_154_aceE	BUsg199_aceE	BU205_aceE	PputGB1_0367_aceE	ACIAD3507_aceE	Shewana3_0426_aceE	STM0152_aceE	pyruvate dehydrogenase, decarboxylase component E1, thiamin-binding
b0115	*aceF*	COG0508C	Bfl152_aceF	BPEN_157_aceF	BVAF_153_aceF	BUsg200_aceF	BU206_aceF	PputGB1_0366_-	ACIAD3506_aceF	Shewana3_0427_-	STM0153_aceF	pyruvate dehydrogenase, dihydrolipoyltransacetylase component E2
b0145	*dksA*	COG1734T	Bfl149_dksA	BPEN_154_dksA	BVAF_150_dksA	BUsg192_dksA	BU198_dksA	PputGB1_4691_-	ACIAD0273_dksA	Shewana3_3404_-	STM0186_dksA	DNA-binding transcriptional regulator of rRNA transcription, DnaK suppressor protein
b0156	*erpA*	COG0316S	Bfl155_yadR	BPEN_160_yadR	BVAF_156_erpA	BUsg205_yadR	BU211_yadR	PputGB1_0463_-	ACIAD0010_-	Shewana3_3067_-	STM0204.S_yadR	conserved protein
b0168	*map*	COG0024J	Bfl270_map	BPEN_278_map	BVAF_273_map	BUsg224_map	BU230_map	PputGB1_1145_-	ACIAD2271_map	Shewana3_2816_-	STM0215_map	methionine aminopeptidase
b0169	*rpsB*	COG0052J	Bfl271_rpsB	BPEN_279_rpsB	BVAF_274_rpsB	BUsg225_rpsB	BU231_rpsB	PputGB1_1146_rpsB	ACIAD2269_rpsB	Shewana3_2815_rpsB	STM0216_rpsB	30S ribosomal subunit protein S2
b0170	*tsf*	COG0264J	Bfl272_tsf	BPEN_280_tsf	BVAF_275_tsf	BUsg226_tsf	BU232_tsf	PputGB1_1147_tsf	ACIAD2268_tsf	Shewana3_2814_tsf	STM0217_tsf	protein chain elongation factor EF-Ts
b0171	*pyrH*	COG0528F	Bfl273_pyrH	BPEN_281_pyrH	BVAF_276_pyrH	BUsg227_pyrH	BU233_pyrH	PputGB1_1148_pyrH	ACIAD1372_pyrH	Shewana3_2813_pyrH	STM0218_pyrH	uridylate kinase
b0172	*frr*	COG0233J	Bfl274_frr	BPEN_282_frr	BVAF_277_frr	BUsg228_frr	BU234_frr	PputGB1_1149_frr	ACIAD1373_frr	Shewana3_2812_frr	STM0219_frr	ribosome recycling factor
b0173	*dxr*	COG0743I	Bfl275_dxr	BPEN_283_dxr	BVAF_278_dxr	BUsg229_dxr	BU235_dxr	PputGB1_1152_-	ACIAD1376_dxr	Shewana3_2809_-	STM0220_dxr	1-deoxy-D-xylulose 5-phosphate reductoisomerase
b0174	*ispU*	COG0020I	Bfl276_uppS	BPEN_284_uppS	BVAF_279_uppS	BUsg230_uppS	BU236_uppS	PputGB1_1150_-	ACIAD1374_ispU	Shewana3_2811_-	STM0221_uppS	undecaprenyl pyrophosphate synthase
b0177	*bamA*	COG4775M	Bfl279_yaeT	BPEN_287_yaeT	BVAF_282_yaeT	BUsg231_yaeT	BU237_yaeT	PputGB1_1154_-	ACIAD1378_-	Shewana3_2807_-	STM0224_yaeT	conserved protein
b0184	*dnaE*	COG0587L	Bfl286_dnaE	BPEN_294_dnaE	BVAF_289_dnaE	BUsg233_dnaE	BU238_dnaE	PputGB1_1161_dnaE	ACIAD2089_dnaE	Shewana3_2800_dnaE	STM0231_dnaE	DNA polymerase III alpha subunit
b0188	*tilS*	COG0037D	Bfl288_mesJ	BPEN_296_tilS	BVAF_291_tilS	BUsg103_mesJ	BU110_mesJ	PputGB1_1163_-	ACIAD3034_mesJ	Shewana3_2799_-	STM0236_tilS	tRNA(Ile)-lysidine synthetase
b0194	*proS*	COG0442J	Bfl289_proS	BPEN_297_proS	BVAF_292_proS	BUsg234_proS	BU239_proS	PputGB1_4213_-	ACIAD0782_proS	Shewana3_1398_-	STM0242_proS	prolyl-tRNA synthetase
b0212	*gloB*	COG0491R	Bfl223_gloB	BPEN_230_gloB	BVAF_224_gloB	BUsg238_gloB	BU246_gloB	PputGB1_3716_-	ACIAD2452_-	Shewana3_2369_-	STM0261_gloB	predicted hydroxyacylglutathione hydrolase
b0406	*tgt*	COG0343J	Bfl230_tgt	BPEN_236_tgt	BVAF_231_tgt	BUsg125_tgt	BU133_tgt	PputGB1_0876_tgt	ACIAD0590_tgt	Shewana3_1435_tgt	STM0405_tgt	tRNA-guanine transglycosylase
b0407	*yajC*	COG1862U	Bfl231_yajC	BPEN_237_yajC	BVAF_232_yajC	BUsg126_yajC	BU134_yajC	PputGB1_0877_yajC	ACIAD0589_-	Shewana3_1436_-	STM0406_yajC	SecYEG protein translocase auxillary subunit
b0414	*ribD*	COG0117H	Bfl234_ribD	BPEN_240_ribD	BVAF_235_ribD	BUsg445_ribD1	BU461_ribD1	PputGB1_0560_-	ACIAD0247_ribD	Shewana3_1096_-	STM0416_ribD	fused diaminohydroxyphosphoribosylaminopyrimidine deaminase and 5-amino-6-(5-phosphoribosylamino) uracil reductase
b0415	*ribE*	COG0054H	Bfl235_ribH	BPEN_241_ribH	BVAF_236_ribH	BUsg443_ribH	BU459_ribH	PputGB1_0563_ribH	ACIAD3571_ribH	Shewana3_1099_ribH	STM0417_ribH	riboflavin synthase beta chain
b0416	*nusB*	COG0781K	Bfl236_nusB	BPEN_242_nusB	BVAF_237_nusB	BUsg447_nusB	BU463_nusB	PputGB1_0564_nusB	ACIAD3572_nusB	Shewana3_1100_nusB	STM0418_nusB	transcription antitermination protein
b0420	*dxs*	COG1154HI	Bfl238_dxs	BPEN_244_dxs	BVAF_239_dxs	BUsg448_dxs	BU464_dxs	PputGB1_0572_-	ACIAD3247_dxs	Shewana3_2901_-	STM0422_dxs	1-deoxyxylulose-5-phosphate synthase, thiamine-requiring, FAD-requiring
b0427	*yajR*	COG2814G	Bfl240_yajR	BPEN_247_yajR	BVAF_241_yajR	BUsg450_yajR	BU466_yajR	PputGB1_0513_-	ACIAD3450_-	Shewana3_3591_-	STM0436.S_yajR	predicted transporter
b0428	*cyoE*	COG0109O	Bfl241_cyoE	BPEN_248_cyoE	BVAF_242_cyoE	BUsg452_cyoE	BU468_cyoE	PputGB1_0853_-	ACIAD2429_cyoE	Shewana3_4035_-	STM0439_cyoE	protoheme IX farnesyltransferase
b0429	*cyoD*	COG3125C	Bfl242_cyoD	BPEN_249_cyoD	BVAF_243_cyoD	BUsg453_cyoD	BU469_cyoD	PputGB1_0852_-	ACIAD2428_cyoD	Shewana3_4034_-	STM0440_cyoD	cytochrome o ubiquinol oxidase subunit IV
b0430	*cyoC*	COG1845C	Bfl243_cyoC	BPEN_250_cyoC	BVAF_244_cyoC	BUsg454_cyoC	BU470_cyoC	PputGB1_0851_-	ACIAD2427_cyoC	Shewana3_4033_-	STM0441_cyoC	cytochrome o ubiquinol oxidase subunit III
b0431	*cyoB*	COG0843C	Bfl244_cyoB	BPEN_251_cyoB	BVAF_245_cyoB	BUsg455_cyoB	BU471_cyoB	PputGB1_0850_-	ACIAD2426_cyoB	Shewana3_4032_-	STM0442_cyoB	cytochrome o ubiquinol oxidase subunit I
b0432	*cyoA*	COG1622C	Bfl245_cyoA	BPEN_252_cyoA	BVAF_246_cyoA	BUsg456_cyoA	BU472_cyoA	PputGB1_0849_-	ACIAD2425_cyoA	Shewana3_4031_-	STM0443_cyoA	cytochrome o ubiquinol oxidase subunit II
b0437	*clpP*	COG0740OU	Bfl246_clpP	BPEN_253_clpP	BVAF_247_clpP	BUsg459_clpP	BU475_clpP	PputGB1_1902_clpP	ACIAD0534_clpP	Shewana3_2661_clpP	STM0448_clpP	proteolytic subunit of ClpA-ClpP and ClpX-ClpP ATP-dependent serine proteases
b0438	*clpX*	COG1219O	Bfl247_clpX	BPEN_254_clpX	BVAF_248_clpX	BUsg460_clpX	BU476_clpX	PputGB1_1903_clpX	ACIAD0535_clpX	Shewana3_2660_clpX	STM0449_clpX	ATPase and specificity subunit of ClpX-ClpP ATP-dependent serine protease
b0439	*lon*	COG0466O	Bfl299_lon	BPEN_307_lon	BVAF_302_lon	BUsg461_lon	BU477_lon	PputGB1_1904_-	ACIAD1115_lon	Shewana3_2659_-	STM0450_lon	DNA-binding ATP-dependent protease La

b0474	*adk*	COG0563F	Bfl302_adk	BPEN_310_adk	BVAF_305_adk	BUsg469_adk	BU484_adk	PputGB1_1111_adk	ACIAD1105_adk	Shewana3_2441_adk	STM0488_adk	adenylate kinase
b0526	*cysS*	COG0215J	Bfl304_cysS	BPEN_312_cysS	BVAF_307_cysS	BUsg471_cysS	BU487_cysS	PputGB1_2878_cysS	ACIAD1481_cysS	Shewana3_2664_cysS	STM0537_cysS	cysteinyl-tRNA synthetase
b0605	*ahpC*	COG0450O	Bfl228_ahpC	BPEN_235_ahpC	BVAF_229_ahpC	BUsg176_ahpC	BU182_ahpC	PputGB1_3534_-	ACIAD2103_ahpC	Shewana3_3325_-	STM0608_ahpC	alkyl hydroperoxide reductase, C22 subunit
b0640	*holA*	COG1466L	Bfl311_holA	BPEN_319_holA	BVAF_314_holA	BUsg430_holA	BU445_holA	PputGB1_4849_holA	ACIAD3108_-	Shewana3_0999_-	STM0646_holA	DNA polymerase III, delta subunit
b0642	*leuS*	COG0495J	Bfl313_leuS	BPEN_321_leuS	BVAF_316_leuS	BUsg429_leuS	BU444_leuS	PputGB1_4847_leuS	ACIAD3106_leuS	Shewana3_1001_leuS	STM0648_leuS	leucyl-tRNA synthetase
b0658	*ybeX*	COG4535P	Bfl315_ybeX	BPEN_323_ybeX	BVAF_318_ybeX	BUsg428_ybeX	BU443_ybeX	PputGB1_4843_-	ACIAD0416_corC	Shewana3_1005_-	STM0667_ybeX	predicteed ion transport
b0659	*ybeY*	COG0319R	Bfl316_ybeY	BPEN_324_ybeY	BVAF_319_ybeY	BUsg427_ybeY	BU442_ybeY	PputGB1_4842_-	ACIAD3160_-	Shewana3_1006_-	STM0668_ybeY	conserved protein
b0680	*glnS*	COG0008J	Bfl324_glnS	BPEN_332_glnS	BVAF_327_glnS	BUsg398_glnS	BU415_glnS	PputGB1_2879_-	ACIAD1920_glnS	Shewana3_2669_-	STM0686_glnS	glutamyl-tRNA synthetase
b0726	*sucA*	COG0567C	Bfl331_sucA	BPEN_341_sucA	BVAF_333_sucA	BUsg292_sucA	BU302_sucA	PputGB1_3760_sucA	ACIAD2876_sucA	Shewana3_1710_sucA	STM0736_sucA	2-oxoglutarate decarboxylase, thiamin-requiring
b0727	*sucB*	COG0508C	Bfl332_sucB	BPEN_342_sucB	BVAF_334_sucB	BUsg293_sucB	BU303_sucB	PputGB1_3759_-	ACIAD2875_sucB	Shewana3_1711_-	STM0737_sucB	dihydrolipoyltranssuccinase
b0884	*infA*	COG0361J	Bfl388_infA	BPEN_399_infA	BVAF_391_infA	BUsg305_infA	BU315_infA	PputGB1_3612_infA	ACIAD0472_infA	Shewana3_1754_infA	STM0953_infA	translation initiation factor IF-1
b0888	*trxB*	COG0492O	Bfl387_trxB	BPEN_398_trxB	BVAF_390_trxB	BUsg304_trxB	BU314_trxB	PputGB1_0820_-	ACIAD0890_trxB	Shewana3_2047_-	STM0958_trxB	thioredoxin reductase, FAD/NAD(P)-binding
b0893	*serS*	COG0172J	Bfl384_serS	BPEN_395_serS	BVAF_387_serS	BUsg303_serS	BU313_serS	PputGB1_3605_-	ACIAD2935_serS	Shewana3_2054_-	STM0963_serS	seryl-tRNA synthetase, also charges selenocysteinyl-tRNA with serine
b0907	*serC*	COG1932HE	Bfl383_serC	BPEN_394_serC	BVAF_386_serC	BUsg302_serC	BU312_serC	PputGB1_1359_-	ACIAD2647_serC	Shewana3_1973_-	STM0977_serC	3-phosphoserine/phosphohydroxythreonine aminotransferase
b0908	*aroA*	COG0128E	Bfl382_aroA	BPEN_393_aroA	BVAF_385_aroA	BUsg301_aroA	BU311_aroA	PputGB1_1361_-	ACIAD2222_-	Shewana3_1975_-	STM0978_aroA	5-enolpyruvylshikimate-3-phosphate synthetase
b0911	*rpsA*	COG0539J	Bfl380_rpsA	BPEN_391_rpsA	BVAF_383_rpsA	BUsg299_rpsA	BU309_rpsA	PputGB1_1363_rpsA	ACIAD2347_rpsA	Shewana3_1977_rpsA	STM0981_rpsA	30S ribosomal subunit protein S1
b0969	*yccK*	COG2920P	Bfl418_yccK	BPEN_430_yccK	BVAF_419_tusE	BUsg451_yccK	BU467_yccK	PputGB1_3601_-	ACIAD1895_-	Shewana3_2056_-	STM1084_yccK	predicted sulfite reductase subunit
b1069	*murJ*	COG0728R	Bfl454_mviN	BPEN_469_mviN	BVAF_455_mviN	BUsg321_mviN	BU333_mviN	PputGB1_0647_-	ACIAD0064_-	Shewana3_3138_-	STM1170_mviN	predicted inner membrane protein
b1084	*rne*	COG1530J	Bfl410_rne	BPEN_422_rne	BVAF_412_rne	BUsg335_rne	BU347_rne	PputGB1_1481_-	ACIAD0438_rne	Shewana3_2567_-	STM1185_rne	fused ribonucleaseE: endoribonuclease/RNA-binding protein/RNA degradosome binding protein
b1089	*rpmF*	COG0333J	Bfl408_rpmF	BPEN_420_rpmF	BVAF_411_rpmF	BUsg337_rpmF	BU349_rpmF	PputGB1_1487_rpmF	ACIAD0869_rpmF	Shewana3_2562_rpmF	STM1191_rpmF	50S ribosomal subunit protein L32
b1093	*fabG*	COG1028IQR	Bfl404_fabG	BPEN_416_fabG	BVAF_407_fabG	BUsg339_fabG	BU351_fabG	PputGB1_1490_fabG	ACIAD0871_fabG	Shewana3_2558_-	STM1195_fabG	3-oxoacyl-[acyl-carrier-protein] reductase
b1098	*tmk*	COG0125F	Bfl401_tmk	BPEN_413_tmk	BVAF_404_tmk	BUsg341_tmk	BU353_tmk	PputGB1_1495_tmk	ACIAD2588_tmk	Shewana3_1766_tmk	STM1200_tmk	thymidylate kinase
b1099	*holB*	COG0470L	Bfl400_holB	BPEN_412_holB	BVAF_403_holB	BUsg342_holB	BU354_holB	PputGB1_1496_-	ACIAD2361_holB	Shewana3_1767_-	STM1201_holB	DNA polymerase III, delta prime subunit
b1100	*ycfH*	COG0084L	Bfl399_ycfH	BPEN_411_ycfH	BVAF_402_ycfH	BUsg343_ycfH	BU355_ycfH	PputGB1_1497_-	ACIAD2359_-	Shewana3_1769_-	STM1202_ycfH	predicted metallodependent hydrolase
b1103	*hinT*	COG0537FGR	Bfl398_ycfF	BPEN_410_ycfF	BVAF_401_hinT	BUsg345_ycfF	BU357_ycfF	PputGB1_0458_-	ACIAD2318_-	Shewana3_2507_-	STM1205_ycfF	purine nucleoside phosphoramidase
b1116	*lolC*	COG4591M	Bfl396_ycfU	BPEN_408_lolC	BVAF_399_lolC	BUsg284_ycfU	BU295_ycfU	PputGB1_1695_-	ACIAD2641_lolC	Shewana3_2287_-	STM1217.S_ycfU	outer membrane-specific lipoprotein transporter subunit
b1117	*lolD*	COG1136V	Bfl395_lolD	BPEN_407_lolD	BVAF_398_lolD	BUsg285_ycfV	BU296_ycfV	PputGB1_1696_-	ACIAD2640_lolD	Shewana3_2286_-	STM1218_lolD	outer membrane-specific lipoprotein transporter subunit
b1131	*purB*	COG0015F	Bfl393_purB	BPEN_405_purB	BVAF_396_purB	BUsg253_purB	BU263_purB	PputGB1_3621_-	ACIAD1219_purB	Shewana3_1745_-	STM1232_purB	adenylosuccinate lyase
b1133	*mnmA*	COG0482J	Bfl392_mnmA	BPEN_404_mnmA	BVAF_395_mnmA	BUsg252_mnmA	BU261_mnmA	PputGB1_3619_mnmA	ACIAD1221_mnmA	Shewana3_1747_mnmA	STM1234.S_mnmA	tRNA (5-methylaminomethyl-2-thiouridylate)-methyltransferase
b1174	*minE*	COG0851D	Bfl441_minE	BPEN_455_minE	BVAF_442_minE	BUsg316_minE	BU325_minE	PputGB1_1324_minE	ACIAD0893_minE	Shewana3_2384_minE	STM1816_minE	cell division topological specificity factor
b1175	*minD*	COG2894D	Bfl440_minD	BPEN_454_minD	BVAF_441_minD	BUsg317_minD	BU326_minD	PputGB1_1325_-	ACIAD0894_minD	Shewana3_2383_-	STM1815_minD	membrane ATPase of the MinC-MinD-MinE system
b1176	*minC*	COG0850D	Bfl439_minC	BPEN_453_minC	BVAF_440_minC	BUsg318_minC	BU327_minC	PputGB1_1326_minC	ACIAD0895_minC	Shewana3_2382_minC	STM1814_minC	cell division inhibitor
b1203	*ychF*	COG0012J	Bfl344_ychF	BPEN_354_ychF	BVAF_346_engD	BUsg185_ychF	BU191_ychF	PputGB1_0763_-	ACIAD2071_-	Shewana3_1012_-	STM1784_ychF	predicted GTP-binding protein
b1204	*pth*	COG0193J	Bfl345_pth	BPEN_355_pth	BVAF_347_pth	BUsg184_pth	BU190_pth	PputGB1_0764_-	ACIAD2909_pth	Shewana3_1011_-	STM1783.S_pth	peptidyl-tRNA hydrolase
b1207	*prs*	COG0462FE	Bfl346_prsA	BPEN_356_prsA	BVAF_348_prs	BUsg163_prsA	BU169_prsA	PputGB1_0766_-	ACIAD2907_prs	Shewana3_0765_-	STM1780_prsA	phosphoribosylpyrophosphate synthase
b1208	*ispE*	COG1947I	Bfl347_ipk	BPEN_357_ispE	BVAF_349_ispE	BUsg164_ipk	BU170_ipk	PputGB1_0767_ipk	ACIAD2903_ipk	Shewana3_0766_ipk	STM1779_ipk	4-diphosphocytidyl-2-C-methylerythritol kinase
b1211	*prfA*	COG0216J	Bfl348_prfA	BPEN_359_prfA	BVAF_351_prfA	BUsg165_prfA	BU171_prfA	PputGB1_0774_prfA	ACIAD2418_prfA	Shewana3_0769_prfA	STM1776_prfA	peptide chain release factor RF-1
b1212	*prmC*	COG2890J	Bfl349_hemK	BPEN_360_hemK	BVAF_352_hemK	BUsg166_hemK	BU172_hemK	PputGB1_0775_-	ACIAD2417_hemK	Shewana3_0770_-	STM1775_hemK	N5-glutamine methyltransferase, modifies release factors RF-1 and RF-2
b1260	*trpA*	COG0159E	Bfl431_trpA	BPEN_443_trpA	BVAF_432_trpA	BUsg266_trpA	BU277_trpA	PputGB1_0097_trpA	ACIAD0642_trpA	Shewana3_1515_trpA	STM1727_trpA	tryptophan synthase, alpha subunit
b1261	*trpB*	COG0133E	Bfl430_trpB	BPEN_442_trpB	BVAF_431_trpB	BUsg267_trpB	BU278_trpB	PputGB1_0098_-	ACIAD0636_trpB	Shewana3_1516_-	STM1726_trpB	tryptophan synthase, beta subunit
b1262	*trpC*	COG0134E	Bfl429_trpC	BPEN_441_trpC	BVAF_430_trpC	BUsg268_trpC	BU279_trpC	PputGB1_0452_trpC	ACIAD2463_trpC	Shewana3_1517_-	STM1725_trpC	fused indole-3-glycerolphosphate synthetase/N-(5-phosphoribosyl)anthranilate isomerase
b1277	*ribA*	COG0807H	Bfl425_ribA	BPEN_437_ribA	BVAF_426_ribA	BUsg261_ribA	BU271_ribA	PputGB1_0568_ribA	ACIAD3249_ribA	Shewana3_2600_ribA	STM1711_ribA	GTP cyclohydrolase II
b1633	*nth*	COG0177L	Bfl372_nth	BPEN_383_nth	BVAF_375_nth	BUsg111_nth	BU119_nth	PputGB1_4320_-	ACIAD1108_nth	Shewana3_2174_-	STM1453_nth	DNA glycosylase and apyrimidinic (AP) lyase (endonuclease III)
b1637	*tyrS*	COG0162J	Bfl371_tyrS	BPEN_382_tyrS	BVAF_374_tyrS	BUsg113_tyrS	BU121_tyrS	PputGB1_0466_-	ACIAD0013_tyrS	Shewana3_3058_-	STM1449_tyrS	tyrosyl-tRNA synthetase
b1652	*rnt*	COG0847L	Bfl368_rnt	BPEN_379_rnt	BVAF_371_rnt	BUsg182_rnt	BU188_rnt	PputGB1_4327_-	ACIAD1149_rnt	Shewana3_2540_-	STM1434_rnt	ribonuclease T (RNase T)
b1654	*grxD*	COG0278O	Bfl367_ydhD	BPEN_378_ydhD	BVAF_370_grxD	BUsg181_ydhD	BU187_ydhD	PputGB1_4331_-	ACIAD1232_-	Shewana3_1640_-	STM1433_ydhD	conserved protein
b1713	*pheT*	COG0072J	Bfl356_pheT	BPEN_367_pheT	BVAF_359_pheT	BUsg122_pheT	BU130_pheT	PputGB1_3476_pheT	ACIAD3041_pheT	Shewana3_1864_pheT	STM1338_pheT	phenylalanine tRNA synthetase, beta subunit
b1714	*pheS*	COG0016J	Bfl355_pheS	BPEN_366_pheS	BVAF_358_pheS	BUsg121_pheS	BU129_pheS	PputGB1_3477_-	ACIAD3042_pheS	Shewana3_1863_pheS	STM1337_pheS	phenylalanine tRNA synthetase, alpha subunit
b1716	*rplT*	COG0292J	Bfl354_rplT	BPEN_365_rplT	BVAF_357_rplT	BUsg120_rplT	BU128_rplT	PputGB1_3478_rplT	ACIAD3046_rplT	Shewana3_2046_rplT	STM1336_rplT	50S ribosomal subunit protein L20
b1717	*rpmI*	COG0291J	Bfl353_rpmI	BPEN_364_rpmI	BVAF_356_rpmI	BUsg119_rpmI	BU127_rpmI	PputGB1_3479_rpmI	ACIAD3047_rpmI	Shewana3_2045_rpmI	STM1335_rpmI	50S ribosomal subunit protein L35
b1718	*infC*	COG0290J	Bfl352_infC	BPEN_363_infC	BVAF_355_infC	BUsg118_infC	BU126_infC	PputGB1_3480_infC	ACIAD3054_infC	Shewana3_2044_infC	STM1334.c_infC	protein chain initiation factor IF-3
b1719	*thrS*	COG0441J	Bfl351_thrS	BPEN_362_thrS	BVAF_354_thrS	BUsg117_thrS	BU125_thrS	PputGB1_3481_thrS	ACIAD3055_thrS	Shewana3_2043_-	STM1333_thrS	threonyl-tRNA synthetase
b1807	*yeaZ*	COG1214O	Bfl442_yeaZ	BPEN_456_yeaZ	BVAF_443_yeaZ	BUsg315_yeaZ	BU324_yeaZ	PputGB1_1112_-	ACIAD0677_-	Shewana3_2391_-	STM1820_yeaZ	predicted peptidase
b1866	*aspS*	COG0173J	Bfl452_aspS	BPEN_467_aspS	BVAF_453_aspS	BUsg306_aspS	BU316_aspS	PputGB1_4205_aspS	ACIAD0609_aspS	Shewana3_1953_aspS	STM1901_aspS	aspartyl-tRNA synthetase
b1876	*argS*	COG0018J	Bfl453_argS	BPEN_468_argS	BVAF_454_argS	BUsg237_argS	BU242_argS	PputGB1_5139_argS	ACIAD0164_argS	Shewana3_3673_argS	STM1909_argS	arginyl-tRNA synthetase
b2019	*hisG*	COG0040E	Bfl462_hisG	BPEN_477_hisG	BVAF_463_hisG	BUsg092_hisG	BU099_hisG	PputGB1_0972_hisG	ACIAD0661_hisG	Shewana3_1854_hisG	STM2071_hisG	ATP phosphoribosyltransferase
b2020	*hisD*	COG0141E	Bfl463_hisD	BPEN_478_hisD	BVAF_464_hisD	BUsg093_hisD	BU100_hisD	PputGB1_0973_hisD	ACIAD0663_hisD	Shewana3_1853_hisD	STM2072_hisD	bifunctional histidinal dehydrogenase/ histidinol dehydrogenase
b2021	*hisC*	COG0079E	Bfl464_hisC	BPEN_479_hisC	BVAF_465_hisC	BUsg094_hisC	BU101_hisC	PputGB1_0974_-	ACIAD0664_hisC	Shewana3_1852_-	STM2073_hisC	histidinol-phosphate aminotransferase
b2022	*hisB*	COG0131E	Bfl465_hisB	BPEN_480_hisB	BVAF_466_hisB	BUsg095_hisB	BU102_hisB	PputGB1_0314_hisB	ACIAD3395_hisB	Shewana3_1851_-	STM2074_hisB	fused histidinol-phosphatase/imidazoleglycerol-phosphate dehydratase
b2023	*hisH*	COG0118E	Bfl466_hisH	BPEN_481_hisH	BVAF_467_hisH	BUsg096_hisH	BU103_hisH	PputGB1_0315_hisH	ACIAD3396_hisH	Shewana3_1850_hisH	STM2075_hisH	imidazole glycerol phosphate synthase, glutamine amidotransferase subunit with HisF

b2024	*hisA*	COG0106E	Bfl467_hisA	BPEN_482_hisA	BVAF_468_hisA	BUsg097_hisA	BU104_hisA	PputGB1_0317_-	ACIAD3398_hisA	Shewana3_1849_-	STM2076_hisA	N-(5'-phospho-L-ribosyl-formimino)-5-amino-1-(5'-phosphoribosyl)-4-imidazolecarboxamide isomerase
b2025	*hisF*	COG0107E	Bfl468_hisF	BPEN_483_hisF	BVAF_469_hisF	BUsg098_hisF	BU105_hisF	PputGB1_0318_-	ACIAD3404_hisF	Shewana3_1848_-	STM2077_hisF	imidazole glycerol phosphate synthase, catalytic subunit with HisH
b2114	*metG*	COG0143J	Bfl471_metG	BPEN_486_metG	BVAF_472_metG	BUsg102_metG	BU109_metG	PputGB1_4315_metG	ACIAD0768_metG	Shewana3_1760_metG	STM2155_metG	methionyl-tRNA synthetase
b2185	*rplY*	COG1825J	Bfl473_rplY	BPEN_488_rplY	BVAF_474_rplY	BUsg131_rplY	BU138_rplY	PputGB1_0765_-	ACIAD2908_rplY	Shewana3_1892_-	STM2224_rplY	50S ribosomal subunit protein L25
b2231	*gyrA*	COG0188L	Bfl476_gyrA	BPEN_492_gyrA	BVAF_477_gyrA	BUsg174_gyrA	BU180_gyrA	PputGB1_1358_-	ACIAD2652_gyrA	Shewana3_1972_-	STM2272_gyrA	DNA gyrase (type II topoisomerase), subunit A
b2235	*nrdB*	COG0208F	Bfl479_nrdB	BPEN_495_nrdB	BVAF_480_nrdB	BUsg172_nrdB	BU178_nrdB	PputGB1_4238_-	ACIAD0722_nrdB	Shewana3_1968_nrdB	STM2278_nrdB	ribonucleoside diphosphate reductase 1, beta subunit, ferritin-like
b2315	*folC*	COG0285H	Bfl494_folC	BPEN_510_folC	BVAF_495_folC	BUsg161_folC	BU167_folC	PputGB1_1531_-	ACIAD0644_folC	Shewana3_1480_-	STM2365_folC	bifunctional folylpolyglutamate synthase/ dihydrofolate synthase
b2318	*truA*	COG0101J	Bfl496_truA	BPEN_512_truA	BVAF_497_truA	BUsg193_truA	BU199_truA	PputGB1_1528_truA	ACIAD0474_truA	Shewana3_1479_truA	STM2368_truA	pseudouridylate synthase I
b2323	*fabB*	COG0304IQ	Bfl498_fabB	BPEN_514_fabB	BVAF_499_fabB	BUsg084_fabB	BU092_fabB	PputGB1_3746_-	ACIAD0879_fabB	Shewana3_1475_-	STM2378_fabB	3-oxoacyl-[acyl-carrier-protein] synthase I
b2329	*aroC*	COG0082E	Bfl500_aroC	BPEN_516_aroC	BVAF_501_aroC	BUsg090_aroC	BU097_aroC	PputGB1_1408_-	ACIAD2028_aroC	Shewana3_1469_-	STM2384_aroC	chorismate synthase
b2400	*gltX*	COG0008J	Bfl504_gltX	BPEN_521_gltX	BVAF_504_gltX	BUsg065_gltX	BU070_gltX	PputGB1_1508_gltX	ACIAD3371_gltX	Shewana3_1418_gltX	STM2415_gltX	glutamyl-tRNA synthetase
b2414	*cysK*	COG0031E	Bfl508_cysK	BPEN_525_cysK	BVAF_508_cysK	BUsg063_cysK	BU066_cysK	PputGB1_4092_-	ACIAD1662_cysK	Shewana3_1561_-	STM2430_cysK	cysteine synthase A, O-acetylserine sulfhydrolase A subunit
b2416	*ptsI*	COG1080G	Bfl510_ptsI	BPEN_527_ptsI	BVAF_510_ptsI	BUsg061_ptsI	BU064_ptsI	PputGB1_0827_-	ACIAD1990_fruB	Shewana3_2257_-	STM2432_ptsI	PEP-protein phosphotransferase of PTS system (enzyme I)
b2472	*dapE*	COG0624E	Bfl517_dapE	BPEN_534_dapE	BVAF_517_dapE	BUsg087_dapE	BU095_dapE	PputGB1_1130_-	ACIAD0791_dapE	Shewana3_1912_-	STM2483_dapE	N-succinyl-diaminopimelate deacylase
b2478	*dapA*	COG0329EM	Bfl518_dapA	BPEN_536_dapA	BVAF_518_dapA	BUsg088_dapA	BU096_dapA	PputGB1_4181_-	ACIAD3585_dapA	Shewana3_1656_-	STM2489_dapA	dihydrodipicolinate synthase
b2511	*der*	COG1160R	Bfl530_engA	BPEN_549_engA	BVAF_531_engA	BUsg582_engA	BU607_engA	PputGB1_0900_engA	ACIAD0565_engA	Shewana3_1233_engA	STM2519_engA	predicted GTP-binding protein
b2514	*hisS*	COG0124J	Bfl531_hisS	BPEN_550_hisS	BVAF_532_hisS	BUsg277_hisS	BU288_hisS	PputGB1_0897_hisS	ACIAD0562_hisS	Shewana3_1230_hisS	STM2522_hisS	histidyl tRNA synthetase
b2530	*iscS*	COG1104E	Bfl534_nifS	BPEN_554_nifS	BVAF_536_iscS	BUsg577_nifS	BU602_yfhO	PputGB1_0885_-	ACIAD1404_iscS	Shewana3_2280_-	STM2543_nifS	cysteine desulfurase (tRNA sulfurtransferase), PLP-dependent
b2533	*suhB*	COG0483G	Bfl535_suhB	BPEN_555_suhB	BVAF_537_suhB	BUsg274_suhB	BU285_suhB	PputGB1_0881_-	ACIAD3246_suhB	Shewana3_2284_-	STM2546_suhB	inositol monophosphatase
b2551	*glyA*	COG0112E	Bfl536_glyA	BPEN_556_glyA	BVAF_538_glyA	BUsg278_glyA	BU289_glyA	PputGB1_0703_glyA	ACIAD2255_glyA	Shewana3_1094_glyA	STM2555_glyA	serine hydroxymethyltransferase
b2559	*tadA*	COG0590FJ	Bfl537_yfhC	BPEN_557_tadA	BVAF_539_tadA	BUsg246_yfhC	BU255_yfhC	PputGB1_1032_-	ACIAD2350_-	Shewana3_1238_-	STM2568_yfhC	tRNA-specific adenosine deaminase
b2567	*rnc*	COG0571K	Bfl540_rnc	BPEN_560_rnc	BVAF_542_rnc	BUsg249_rnc	BU258_rnc	PputGB1_4374_rnc	ACIAD2581_rnc	Shewana3_3026_rnc	STM2581_rnc	RNase III
b2568	*lepB*	COG0681U	Bfl541_lepB	BPEN_561_lepB	BVAF_543_lepB	BUsg250_lepB	BU259_lepB	PputGB1_4375_-	ACIAD2583_lepB	Shewana3_3027_-	STM2582_lepB	leader peptidase (signal peptidase I)
b2569	*lepA*	COG0481M	Bfl542_lepA	BPEN_562_lepA	BVAF_544_lepA	BUsg251_lepA	BU260_lepA	PputGB1_4376_-	ACIAD2584_lepA	Shewana3_3028_-	STM2583_lepA	GTP-binding membrane protein
b2595	*bamD*	COG4105R	Bfl180_yfiO	BPEN_186_yfiO	BVAF_181_yfiO	BUsg389_yfiO	BU402_yfiO	PputGB1_0668_-	ACIAD2898_-	Shewana3_3172_-	STM2663_yfiO	predicted lipoprotein
b2599	*pheA*	COG0077E	Bfl179_pheA	BPEN_185_pheA	BVAF_180_pheA	BUsg379_pheA	BU392_pheA	PputGB1_1360_-	ACIAD2223_pheA	Shewana3_3007_-	STM2667_pheA	fused chorismate mutase P/prephenate dehydratase
b2606	*rplS*	COG0335J	Bfl176_rplS	BPEN_182_rplS	BVAF_177_rplS	BUsg384_rplS	BU397_rplS	PputGB1_1070_rplS	ACIAD3310_rplS	Shewana3_3014_rplS	STM2673_rplS	50S ribosomal subunit protein L19
b2607	*trmD*	COG0336J	Bfl175_trmD	BPEN_181_trmD	BVAF_176_trmD	BUsg383_trmD	BU396_trmD	PputGB1_1069_trmD	ACIAD3311_trmD	Shewana3_3015_trmD	STM2674_trmD	tRNA m(1)G37 methyltransferase, SAM-dependent
b2608	*rimM*	COG0806J	Bfl174_rimM	BPEN_180_rimM	BVAF_175_rimM	BUsg382_rimM	BU395_rimM	PputGB1_1068_rimM	ACIAD3312_rimM	Shewana3_3016_rimM	STM2675_rimM	16S rRNA processing protein
b2609	*rpsP*	COG0228J	Bfl173_rpsP	BPEN_179_rpsP	BVAF_174_rpsP	BUsg381_rpsP	BU394_rpsP	PputGB1_1067_rpsP	ACIAD3313_rpsP	Shewana3_3017_rpsP	STM2676_rpsP	30S ribosomal subunit protein S16
b2614	*grpE*	COG0576O	Bfl544_grpE	BPEN_564_grpE	BVAF_546_grpE	BUsg178_grpE	BU184_grpE	PputGB1_4729_-	ACIAD3652_grpE	Shewana3_2902_-	STM2681_grpE	heat shock protein
b2615	*nadK*	COG0061G	Bfl545_ppnK	BPEN_565_ppnK	BVAF_547_ppnK	BUsg179_ppnK	BU185_ppnK	PputGB1_1544_ppnK	ACIAD2231_ppnK	Shewana3_2903_ppnK	STM2683_ppnK	NAD kinase
b2620	*smpB*	COG0691O	Bfl548_smpB	BPEN_568_smpB	BVAF_550_smpB	BUsg245_smpB	BU254_smpB	PputGB1_4734_smpB	ACIAD2894_smpB	Shewana3_2952_smpB	STM2688_smpB	trans-translation protein
b2696	*csrA*	COG1551T	Bfl169_csrA	BPEN_175_csrA	BVAF_171_csrA	BUsg391_csrA	BU404_csrA	PputGB1_3977_-	ACIAD1251_csrA	Shewana3_1130_-	STM2826_csrA	pleiotropic regulatory protein for carbon source metabolism
b2697	*alaS*	COG0013J	Bfl168_alaS	BPEN_174_alaS	BVAF_170_alaS	BUsg390_alaS	BU403_alaS	PputGB1_3979_alaS	ACIAD1253_alaS	Shewana3_1128_alaS	STM2827_alaS	alanyl-tRNA synthetase
b2748	*ftsB*	COG2919D	Bfl165_ygbQ	BPEN_170_ygbQ	BVAF_166_ftsB	BUsg406_ygbQ	BU421_ygbQ	PputGB1_1167_-	ACIAD2000_-	Shewana3_1117_-	STM2931_ftsB	cell division protein
b2764	*cysJ*	COG0369P	Bfl158_cysJ	BPEN_163_cysJ	BVAF_159_cysJ	BUsg413_cysJ	BU428_cysJ	PputGB1_1302_-	ACIAD1908_nasA	Shewana3_0855_-	STM2948_cysJ	sulfite reductase, alpha subunit, flavoprotein
b2779	*eno*	COG0148G	Bfl157_eno	BPEN_162_eno	BVAF_158_eno	BUsg400_eno	BU417_eno	PputGB1_1166_eno	ACIAD2001_eno	Shewana3_1116_eno	STM2952_eno	enolase
b2780	*pyrG*	COG0504F	Bfl156_pyrG	BPEN_161_pyrG	BVAF_157_pyrG	BUsg399_pyrG	BU416_pyrG	PputGB1_1164_pyrG	ACIAD2003_pyrG	Shewana3_1115_pyrG	STM2953_pyrG	CTP synthetase
b2819	*recD*	COG0507L	Bfl267_recD	BPEN_275_recD	BVAF_270_recD	BUsg440_recD	BU455_recD	PputGB1_4670_-	ACIAD0399_recD	Shewana3_2247_-	STM2993_recD	exonuclease V (RecBCD complex), alpha chain
b2820	*recB*	COG1074L	Bfl268_recB	BPEN_276_recB	BVAF_271_recB	BUsg439_recB	BU454_recB	PputGB1_4671_-	ACIAD0398_recB	Shewana3_2246_-	STM2994_recB	exonuclease V (RecBCD complex), beta subunit
b2822	*recC*	COG1330L	Bfl266_recC	BPEN_274_recC	BVAF_269_recC	BUsg438_recC	BU453_recC	PputGB1_4672_-	ACIAD0397_recC	Shewana3_2245_-	STM2996_recC	exonuclease V (RecBCD complex), gamma chain
b2898	*ygfZ*	COG0354R	Bfl260_ygfZ	BPEN_268_ygfZ	BVAF_263_ygfZ	BUsg420_ygfZ	BU435_ygfZ	PputGB1_4388_-	ACIAD0714_-	Shewana3_3417_-	STM3048_ygfZ	predicted folate-dependent regulatory protein
b2914	*rpiA*	COG0120G	Bfl256_rpiA	BPEN_263_rpiA	BVAF_258_rpiA	BUsg397_rpiA	BU411_rpiA	PputGB1_5203_-	ACIAD1358_rpiA	Shewana3_0978_-	STM3063_rpiA	ribose 5-phosphate isomerase, constitutive
b2926	*pgk*	COG0126G	Bfl254_pgk	BPEN_261_pgk	BVAF_256_pgk	BUsg435_pgk	BU450_pgk	PputGB1_5012_pgk	ACIAD1927_pgk	Shewana3_3351_pgk	STM3069_pgk	phosphoglycerate kinase
b2935	*tktA*	COG0021G	Bfl516_tktA	BPEN_533_tktA	BVAF_516_tktA	BUsg086_tkt	BU094_tktB	PputGB1_5014_-	ACIAD2035_tkt	Shewana3_3353_-	STM3076_tktA	transketolase 1, thiamin-binding
b2942	*metK*	COG0192H	Bfl252_metK	BPEN_259_metK	BVAF_254_metK	BUsg393_metK	BU408_metK	PputGB1_5016_-	ACIAD2037_metK	Shewana3_3354_-	STM3090_metK	methionine adenosyltransferase 1
b2949	*yqgF*	COG0816L	Bfl250_yqgF	BPEN_257_yqgF	BVAF_252_yqgF	BUsg530_yqgF	BU548_yqgF	PputGB1_5046_-	ACIAD0352_-	Shewana3_1199_-	STM3097_yqgF	predicted Holliday junction resolvase
b2961	*mutY*	COG1194L	Bfl249_mutY	BPEN_256_mutY	BVAF_250_mutY	BUsg534_mutY	BU552_mutY	PputGB1_0311_-	ACIAD3606_mutY	Shewana3_1181_-	STM3110_mutY	adenine DNA glycosylase
b2962	*yggX*	COG2924CO	Bfl248_yggX	BPEN_255_yggX	BVAF_249_yggX	BUsg535_yggX	BU553_yggX	PputGB1_0310_-	ACIAD0282_-	Shewana3_1180_-	STM3111_yggX	protein that protects iron-sulfur proteins against oxidative damage
b3041	*ribB*	COG0108H	Bfl065_ribB	BPEN_067_ribB	BVAF_065_ribB	BUsg056_ribB	BU059_ribB	PputGB1_0575_ribB	ACIAD2913_ribB	Shewana3_0134_ribB	STM3195_ribB	3,4-dihydroxy-2-butanone-4-phosphate synthase
b3056	*cca*	COG0617J	Bfl062_cca	BPEN_064_cca	BVAF_062_cca	BUsg058_cca	BU061_cca	PputGB1_0425_cca	ACIAD2288_cca	Shewana3_3076_-	STM3204_cca	fused tRNA nucleotidyl transferase/2'3'-cyclic phosphodiesterase/2'nucleotidase and phosphatase
b3064	*ygjD*	COG0533O	Bfl059_gcp	BPEN_060_gcp	BVAF_059_gcp	BUsg055_gcp	BU058_ygjD	PputGB1_0421_-	ACIAD1332_-	Shewana3_3083_-	STM3208_gcp	predicted peptidase
b3065	*rpsU*	COG0828J	Bfl058_rpsU	BPEN_059_rpsU	BVAF_058_rpsU	BUsg054_rpsU	BU057_rpsU	PputGB1_0420_rpsU	ACIAD1331_rpsU	Shewana3_3084_rpsU	STM3209_rpsU	30S ribosomal subunit protein S21
b3066	*dnaG*	COG0358L	Bfl057_dnaG	BPEN_058_dnaG	BVAF_057_dnaG	BUsg053_dnaG	BU056_dnaG	PputGB1_0419_dnaG	ACIAD2899_dnaG	Shewana3_3086_-	STM3210_dnaG	DNA primase
b3067	*rpoD*	COG0568K	Bfl056_rpoD	BPEN_057_rpoD	BVAF_056_rpoD	BUsg052_rpoD	BU055_rpoD	PputGB1_0418_-	ACIAD2925_rpoD	Shewana3_3087_-	STM3211.S_rpoD	RNA polymerase, sigma 70 (sigma D) factor
b3146	*yraL*	COG0313R	Bfl052_yraL	BPEN_053_yraL	BVAF_052_yraL	BUsg083_yraL	BU091_yraL	PputGB1_4522_-	ACIAD1130_-	Shewana3_3879_-	STM3263_yraL	predicted methyltransferase
b3164	*pnp*	COG1185J	Bfl108_pnp	BPEN_112_pnp	BVAF_109_pnp	BUsg361_pnp	BU373_pnp	PputGB1_4707_-	ACIAD0402_pnp	Shewana3_1035_-	STM3282_pnp	polynucleotide phosphorylase/polyadenylase
b3165	*rpsO*	COG0184J	Bfl107_rpsO	BPEN_111_rpsO	BVAF_108_rpsO	BUsg362_rpsO	BU374_rpsO	PputGB1_4709_rpsO	ACIAD0401_rpsO	Shewana3_1033_rpsO	STM3283_rpsO	30S ribosomal subunit protein S15
b3166	*truB*	COG0130J	Bfl106_truB	BPEN_110_truB	BVAF_107_truB	BUsg363_truB	BU375_truB	PputGB1_4710_-	ACIAD3307_truB	Shewana3_1032_truB	STM3284_truB	tRNA U55 pseudouridine synthase

b3167	*rbfA*	COG0858J	Bfl105_rbfA	BPEN_109_rbfA	BVAF_106_rbfA	BUsg364_rbfA	BU376_rbfA	PputGB1_4711_rbfA	ACIAD0370_rbfA	Shewana3_1031_rbfA	STM3285_rbfA	30s ribosome binding factor
b3168	*infB*	COG0532J	Bfl104_infB	BPEN_108_infB	BVAF_105_infB	BUsg365_infB	BU377_infB	PputGB1_4712_infB	ACIAD0369_infB	Shewana3_1030_infB	STM3286_infB	fused protein chain initiation factor 2, IF2: membrane protein/conserved protein
b3169	*nusA*	COG0195K	Bfl103_nusA	BPEN_107_nusA	BVAF_104_nusA	BUsg366_nusA	BU378_nusA	PputGB1_4713_nusA	ACIAD0368_nusA	Shewana3_1029_nusA	STM3287_nusA	transcription termination/antitermination L factor
b3178	*ftsH*	COG0465O	Bfl098_ftsH	BPEN_101_hflB	BVAF_098_ftsH	BUsg369_ftsH	BU382_hflB	PputGB1_4719_-	ACIAD2853_ftsH	Shewana3_1023_-	STM3296_hflB	protease, ATP-dependent zinc-metallo
b3179	*rlmE*	COG0293J	Bfl097_ftsJ	BPEN_100_rrmJ	BVAF_097_rlmE	BUsg370_ftsJ	BU383_ftsJ	PputGB1_4720_-	ACIAD2854_ftsJ	Shewana3_1022_rrmJ	STM3297_rrmJ	23S rRNA methyltransferase
b3181	*greA*	COG0782K	Bfl096_greA	BPEN_099_greA	BVAF_096_greA	BUsg371_greA	BU384_greA	PputGB1_4723_greA	ACIAD2862_greA	Shewana3_1017_-	STM3299_greA	transcription elongation factor
b3183	*obgE*	COG0536R	Bfl095_yhbZ	BPEN_098_obgE	BVAF_095_obgE	BUsg376_obgE	BU389_obgE	PputGB1_0722_obgE	ACIAD2561_obgE	Shewana3_3217_obgE	STM3301_obgE	GTPase involved in cell partioning and DNA repair
b3185	*rpmA*	COG0211J	Bfl094_rpmA	BPEN_097_rpmA	BVAF_094_rpmA	BUsg375_rpmA	BU388_rpmA	PputGB1_0721_rpmA	ACIAD2938_rpmA	Shewana3_3218_rpmA	STM3303_rpmA	50S ribosomal subunit protein L27
b3186	*rplU*	COG0261J	Bfl093_rplU	BPEN_096_rplU	BVAF_093_rplU	BUsg374_rplU	BU387_rplU	PputGB1_0720_rplU	ACIAD2939_rplU	Shewana3_3219_rplU	STM3304_rplU	50S ribosomal subunit protein L21
b3189	*murA*	COG0766M	Bfl046_murA	BPEN_047_murA	BVAF_046_murA	BUsg373_murA	BU386_murA	PputGB1_0971_-	ACIAD0660_murA	Shewana3_0684_-	STM3307_murA	UDP-N-acetylglucosamine 1-carboxyvinyltransferase
b3190	*yrbA*	COG5007K	Bfl045_yrbA	BPEN_046_yrbA	BVAF_045_yrbA	BUsg372_yrbA	BU385_yrbA	PputGB1_0970_-	ACIAD0659_-	Shewana3_0683_-	STM3308_yrbA	predicted DNA-binding transcriptional regulator
b3230	*rpsI*	COG0103J	Bfl050_rpsI	BPEN_051_rpsI	BVAF_050_rpsI	BUsg377_rpsI	BU390_rpsI	PputGB1_4533_rpsI	ACIAD3013_rpsI	Shewana3_0693_rpsI	STM3344_rpsI	30S ribosomal subunit protein S9
b3231	*rplM*	COG0102J	Bfl049_rplM	BPEN_050_rplM	BVAF_049_rplM	BUsg378_rplM	BU391_rplM	PputGB1_4534_rplM	ACIAD3012_rplM	Shewana3_0692_rplM	STM3345_rplM	50S ribosomal subunit protein L13
b3281	*aroE*	COG0169E	Bfl221_aroE	BPEN_228_aroE	BVAF_222_aroE	BUsg474_aroE	BU493_aroE	PputGB1_0090_aroE	ACIAD0418_aroE	Shewana3_0043_aroE	STM3401_aroE	dehydroshikimate reductase, NAD(P)-binding
b3282	*rimN*	COG0009J	Bfl220_yrdC	BPEN_227_yrdC	BVAF_221_yrdC	BUsg475_yrdC	BU494_yrdC	PputGB1_0086_-	ACIAD0208_-	Shewana3_0040_-	STM3402_yrdC	predicted ribosome maturation factor
b3287	*def*	COG0242J	Bfl219_def	BPEN_226_def	BVAF_220_def	BUsg477_def	BU496_def	PputGB1_0084_def	ACIAD0211_def	Shewana3_2319_def	STM3406_def	peptide deformylase
b3288	*fmt*	COG0223J	Bfl218_fmt	BPEN_225_fmt	BVAF_219_fmt	BUsg478_fmt	BU497_fmt	PputGB1_0083_fmt	ACIAD3637_fmt	Shewana3_0034_-	STM3407_fmt	10-formyltetrahydrofolate:L-methionyl-tRNA(fMet) N-formyltransferase
b3294	*rplQ*	COG0203J	Bfl217_rplQ	BPEN_224_rplQ	BVAF_218_rplQ	BUsg479_rplQ	BU498_rplQ	PputGB1_0509_rplQ	ACIAD3193_rplQ	Shewana3_0225_rplQ	STM3414_rplQ	50S ribosomal subunit protein L17
b3295	*rpoA*	COG0202K	Bfl216_rpoA	BPEN_223_rpoA	BVAF_217_rpoA	BUsg480_rpoA	BU499_rpoA	PputGB1_0508_-	ACIAD3194_rpoA	Shewana3_0224_-	STM3415_rpoA	RNA polymerase, alpha subunit
b3296	*rpsD*	COG0522J	Bfl215_rpsD	BPEN_222_rpsD	BVAF_216_rpsD	BUsg481_rpsD	BU500_rpsD	PputGB1_0507_rpsD	ACIAD3195_rpsD	Shewana3_0223_rpsD	STM3416_rpsD	30S ribosomal subunit protein S4
b3297	*rpsK*	COG0100J	Bfl214_rpsK	BPEN_221_rpsK	BVAF_215_rpsK	BUsg482_rpsK	BU501_rpsK	PputGB1_0506_-	ACIAD3196_rpsK	Shewana3_0222_-	STM3417_rpsK	30S ribosomal subunit protein S11
b3298	*rpsM*	COG0099J	Bfl213_rpsM	BPEN_220_rpsM	BVAF_214_rpsM	BUsg483_rpsM	BU502_rpsM	PputGB1_0505_rpsM	ACIAD3197_rpsM	Shewana3_0221_rpsM	STM3418_rpsM	30S ribosomal subunit protein S13
b3300	*secY*	COG0201U	Bfl211_secY	BPEN_218_secY	BVAF_212_secY	BUsg485_secY	BU504_secY	PputGB1_0504_secY	ACIAD3199_secY	Shewana3_0219_secY	STM3420_secY	preprotein translocase membrane subunit
b3301	*rplO*	COG0200J	Bfl210_rplO	BPEN_217_rplO	BVAF_211_rplO	BUsg486_rplO	BU505_rplO	PputGB1_0503_rplO	ACIAD3200_rplO	Shewana3_0218_rplO	STM3421_rplO	50S ribosomal subunit protein L15
b3303	*rpsE*	COG0098J	Bfl208_rpsE	BPEN_215_rpsE	BVAF_209_rpsE	BUsg488_rpsE	BU507_rpsE	PputGB1_0501_rpsE	ACIAD3202_rpsE	Shewana3_0216_rpsE	STM3423_rpsE	30S ribosomal subunit protein S5
b3304	*rplR*	COG0256J	Bfl207_rplR	BPEN_214_rplR	BVAF_208_rplR	BUsg489_rplR	BU508_rplR	PputGB1_0500_rplR	ACIAD3203_rplR	Shewana3_0215_rplR	STM3424_rplR	50S ribosomal subunit protein L18
b3305	*rplF*	COG0097J	Bfl206_rplF	BPEN_213_rplF	BVAF_207_rplF	BUsg490_rplF	BU509_rplF	PputGB1_0499_rplF	ACIAD3204_rplF	Shewana3_0214_rplF	STM3425_rplF	50S ribosomal subunit protein L6
b3306	*rpsH*	COG0096J	Bfl205_rpsH	BPEN_212_rpsH	BVAF_206_rpsH	BUsg491_rpsH	BU510_rpsH	PputGB1_0498_rpsH	ACIAD3205_rpsH	Shewana3_0213_rpsH	STM3426_rpsH	30S ribosomal subunit protein S8
b3307	*rpsN*	COG0199J	Bfl204_rpsN	BPEN_211_rpsN	BVAF_205_rpsN	BUsg492_rpsN	BU511_rpsN	PputGB1_0497_rpsN	ACIAD3206_rpsN	Shewana3_0212_rpsN	STM3427.S_rpsN	30S ribosomal subunit protein S14
b3308	*rplE*	COG0094J	Bfl203_rplE	BPEN_210_rplE	BVAF_204_rplE	BUsg493_rplE	BU512_rplE	PputGB1_0496_rplE	ACIAD3207_rplE	Shewana3_0211_rplE	STM3428_rplE	50S ribosomal subunit protein L5
b3309	*rplX*	COG0198J	Bfl202_rplX	BPEN_209_rplX	BVAF_203_rplX	BUsg494_rplX	BU513_rplX	PputGB1_0495_rplX	ACIAD3208_rplX	Shewana3_0210_rplX	STM3429_rplX	50S ribosomal subunit protein L24
b3310	*rplN*	COG0093J	Bfl201_rplN	BPEN_208_rplN	BVAF_202_rplN	BUsg495_rplN	BU514_rplN	PputGB1_0494_rplN	ACIAD3209_rplN	Shewana3_0209_rplN	STM3430_rplN	50S ribosomal subunit protein L14
b3311	*rpsQ*	COG0186J	Bfl200_rpsQ	BPEN_207_rpsQ	BVAF_201_rpsQ	BUsg496_rpsQ	BU515_rpsQ	PputGB1_0493_rpsQ	ACIAD3210_rpsQ	Shewana3_0208_rpsQ	STM3431_rpsQ	30S ribosomal subunit protein S17
b3313	*rplP*	COG0197J	Bfl198_rplP	BPEN_205_rplP	BVAF_199_rplP	BUsg498_rplP	BU517_rplP	PputGB1_0491_rplP	ACIAD3212_rplP	Shewana3_0206_rplP	STM3433_rplP	50S ribosomal subunit protein L16
b3314	*rpsC*	COG0092J	Bfl197_rpsC	BPEN_204_rpsC	BVAF_198_rpsC	BUsg499_rpsC	BU518_rpsC	PputGB1_0490_rpsC	ACIAD3213_rpsC	Shewana3_0205_rpsC	STM3434_rpsC	30S ribosomal subunit protein S3
b3315	*rplV*	COG0091J	Bfl196_rplV	BPEN_203_rplV	BVAF_197_rplV	BUsg500_rplV	BU519_rplV	PputGB1_0489_rplV	ACIAD3214_rplV	Shewana3_0204_rplV	STM3435_rplV	50S ribosomal subunit protein L22
b3316	*rpsS*	COG0185J	Bfl195_rpsS	BPEN_202_rpsS	BVAF_196_rpsS	BUsg501_rpsS	BU520_rpsS	PputGB1_0488_rpsS	ACIAD3215_rpsS	Shewana3_0203_rpsS	STM3436_rpsS	30S ribosomal subunit protein S19
b3317	*rplB*	COG0090J	Bfl194_rplB	BPEN_201_rplB	BVAF_195_rplB	BUsg502_rplB	BU521_rplB	PputGB1_0487_rplB	ACIAD3216_rplB	Shewana3_0202_rplB	STM3437_rplB	50S ribosomal subunit protein L2
b3318	*rplW*	COG0089J	Bfl193_rplW	BPEN_200_rplW	BVAF_194_rplW	BUsg503_rplW	BU522_rplW	PputGB1_0486_rplW	ACIAD3217_rplW	Shewana3_0201_rplW	STM3438_rplW	50S ribosomal subunit protein L23
b3319	*rplD*	COG0088J	Bfl192_rplD	BPEN_199_rplD	BVAF_193_rplD	BUsg504_rplD	BU523_rplD	PputGB1_0485_rplD	ACIAD3218_rplD	Shewana3_0200_rplD	STM3439_rplD	50S ribosomal subunit protein L4
b3320	*rplC*	COG0087J	Bfl191_rplC	BPEN_198_rplC	BVAF_192_rplC	BUsg505_rplC	BU524_rplC	PputGB1_0484_rplC	ACIAD3219_rplC	Shewana3_0199_rplC	STM3440_rplC	50S ribosomal subunit protein L3
b3321	*rpsJ*	COG0051J	Bfl190_rpsJ	BPEN_197_rpsJ	BVAF_191_rpsJ	BUsg506_rpsJ	BU525_rpsJ	PputGB1_0483_rpsJ	ACIAD3220_rpsJ	Shewana3_0198_rpsJ	STM3441_rpsJ	30S ribosomal subunit protein S10
b3341	*rpsG*	COG0049J	Bfl566_rpsG	BPEN_586_rpsG	BVAF_568_rpsG	BUsg509_rpsG	BU528_rpsG	PputGB1_0480_-	ACIAD0883_rpsG	Shewana3_0195_-	STM3447_rpsG	30S ribosomal subunit protein S7
b3342	*rpsL*	COG0048J	Bfl567_rpsL	BPEN_587_rpsL	BVAF_569_rpsL	BUsg510_rpsL	BU529_rpsL	PputGB1_0479_rpsL	ACIAD0881_rpsL	Shewana3_0194_rpsL	STM3448_rpsL	30S ribosomal subunit protein S12
b3384	*trpS*	COG0180J	Bfl569_trpS	BPEN_589_trpS	BVAF_571_trpS	BUsg517_trpS	BU536_trpS	PputGB1_4538_-	ACIAD2871_trpS	Shewana3_3886_-	STM3481_trpS	tryptophanyl-tRNA synthetase
b3386	*rpe*	COG0036G	Bfl570_rpe	BPEN_590_rpe	BVAF_572_rpe	BUsg518_rpe	BU537_rpe	PputGB1_0446_-	ACIAD0685_rpe	Shewana3_3888_-	STM3483_rpe	D-ribulose-5-phosphate 3-epimerase
b3389	*aroB*	COG0337E	Bfl571_aroB	BPEN_591_aroB	BVAF_573_aroB	BUsg519_aroB	BU538_aroB	PputGB1_5128_aroB	ACIAD3353_aroB	Shewana3_3893_aroB	STM3486_aroB	3-dehydroquinate synthase
b3390	*aroK*	COG0703E	Bfl572_aroK	BPEN_592_aroK	BVAF_574_aroK	BUsg520_aroK	BU539_aroK	PputGB1_5129_aroK	ACIAD3354_aroK	Shewana3_3894_aroK	STM3487_aroK	shikimate kinase I
b3414	*nfuA*	COG0694O	Bfl573_yhgI	BPEN_593_gntY	BVAF_575_nfuA	BUsg525_yhgI	BU544_yhgI	PputGB1_1985_-	ACIAD1052_-	Shewana3_4003_-	STM3511_yhgI	predicted gluconate transport associated protein
b3461	*rpoH*	COG0568K	Bfl626_rpoH	BPEN_653_rpoH	BVAF_627_rpoH	BUsg026_rpoH	BU025_rpoH	PputGB1_5158_-	ACIAD1311_rpoH	Shewana3_3969_-	STM3568_rpoH	RNA polymerase, sigma 32 (sigma H) factor
b3559	*glyS*	COG0751J	Bfl021_glyS	BPEN_020_glyS	BVAF_020_glyS	BUsg127_glyS	BU135_glyS	PputGB1_0076_glyS	ACIAD3269_glyS	Shewana3_0016_glyS	STM3655_glyS	glycine tRNA synthetase, beta subunit
b3560	*glyQ*	COG0752J	Bfl020_glyQ	BPEN_019_glyQ	BVAF_019_glyQ	BUsg128_glyQ	BU136_glyQ	PputGB1_0077_glyQ	ACIAD3268_glyQ	Shewana3_0017_glyQ	STM3656_glyQ	glycine tRNA synthetase, alpha subunit
b3607	*cysE*	COG1045E	Bfl603_cysE	BPEN_625_cysE	BVAF_606_cysE	BUsg051_cysE	BU054_cysE	PputGB1_0883_-	ACIAD2091_cysE	Shewana3_2282_-	STM3699_cysE	serine acetyltransferase
b3611	*yibN*	COG0607P	Bfl606_yibN	BPEN_629_yibN	BVAF_609_yibN	BUsg049_yibN	BU052_yibN	PputGB1_5106_-	ACIAD3120_-	Shewana3_0052_-	STM3703_yibN	predicted rhodanese-related sulfurtransferase
b3636	*rpmG*	COG0267J	Bfl611_rpmG	BPEN_635_rpmG	BVAF_613_rpmG	BUsg078_rpmG	BU085_rpmG	PputGB1_5332_rpmG	ACIAD0501_rpmG	Shewana3_3768_rpmG	STM3727_rpmG	50S ribosomal subunit protein L33
b3637	*rpmB*	COG0227J	Bfl612_rpmB	BPEN_636_rpmB	BVAF_614_rpmB	BUsg079_rpmB	BU086_rpmB	PputGB1_5333_rpmB	ACIAD0502_rpmB	Shewana3_3769_rpmB	STM3728_rpmB	50S ribosomal subunit protein L28
b3640	*dut*	COG0756F	Bfl613_dut	BPEN_638_dut	BVAF_615_dut	BUsg540_dut	BU560_dut	PputGB1_5337_dut	ACIAD0901_dut	Shewana3_3772_dut	STM3731_dut	deoxyuridinetriphosphatase
b3648	*gmk*	COG0194F	Bfl616_gmk	BPEN_641_gmk	BVAF_618_gmk	BUsg419_gmk	BU434_gmk	PputGB1_5344_gmk	ACIAD3324_gmk	Shewana3_3810_gmk	STM3740_gmk	guanylate kinase
b3699	*gyrB*	COG0187L	Bfl017_gyrB	BPEN_016_gyrB	BVAF_016_gyrB	BUsg010_gyrB	BU010_gyrB	PputGB1_0006_gyrB	ACIAD0004_gyrB	Shewana3_0012_-	STM3835_gyrB	DNA gyrase, subunit B
b3701	*dnaN*	COG0592L	Bfl016_dnaN	BPEN_015_dnaN	BVAF_015_dnaN	BUsg011_dnaN	BU011_dnaN	PputGB1_0004_-	ACIAD0002_dnaN	Shewana3_0010_-	STM3837_dnaN	DNA polymerase III, beta subunit

b3704	*rnpA*	COG0594J	Bfl014_rnpA	BPEN_013_rnpA	BVAF_013_rnpA	BUsg014_rnpA	BU014_rnpA	PputGB1_0001_rnpA	ACIAD3683_rnpA	Shewana3_0008_rnpA	STM3840_rnpA	protein C5 component of RNase P
b3706	*mnmE*	COG0486R	Bfl011_trmE	BPEN_011_trmE	BVAF_011_mnmE	BUsg017_trmE	BU016_trmE	PputGB1_5443_trmE	ACIAD3680_trmE	Shewana3_0005_trmE	STM3843_trmE	GTPase
b3730	*glmU*	COG1207M	Bfl010_glmU	BPEN_010_glmU	BVAF_010_glmU	BUsg028_glmU	BU027_glmU	PputGB1_5429_-	ACIAD3575_glmU	Shewana3_4128_-	STM3862_glmU	fused N-acetyl glucosamine-1-phosphate uridyltransferase/glucosamine-1-phosphate acetyl transferase
b3731	*atpC*	COG0355C	Bfl009_atpC	BPEN_009_atpC	BVAF_009_atpC	BUsg009_atpC	BU009_atpC	PputGB1_5430_atpC	ACIAD0188_atpC	Shewana3_4129_atpC	STM3864_atpC	F1 sector of membrane-bound ATP synthase, epsilon subunit
b3732	*atpD*	COG0055C	Bfl008_atpD	BPEN_008_atpD	BVAF_008_atpD	BUsg008_atpD	BU008_atpD	PputGB1_5431_-	ACIAD0187_atpD	Shewana3_4130_-	STM3865_atpD	F1 sector of membrane-bound ATP synthase, beta subunit
b3733	*atpG*	COG0224C	Bfl007_atpG	BPEN_007_atpG	BVAF_007_atpG	BUsg007_atpG	BU007_atpG	PputGB1_5432_-	ACIAD0186_atpG	Shewana3_4131_-	STM3866_atpG	F1 sector of membrane-bound ATP synthase, gamma subunit
b3734	*atpA*	COG0056C	Bfl006_atpA	BPEN_006_atpA	BVAF_006_atpA	BUsg006_atpA	BU006_atpA	PputGB1_5433_-	ACIAD0185_atpA	Shewana3_4132_-	STM3867_atpA	F1 sector of membrane-bound ATP synthase, alpha subunit
b3735	*atpH*	COG0712C	Bfl005_atpH	BPEN_005_atpH	BVAF_005_atpH	BUsg005_atpH	BU005_atpH	PputGB1_5434_-	ACIAD0184_atpH	Shewana3_4133_-	STM3868_atpH	F1 sector of membrane-bound ATP synthase, delta subunit
b3736	*atpF*	COG0711C	Bfl004_atpF	BPEN_004_atpF	BVAF_004_atpF	BUsg004_atpF	BU004_atpF	PputGB1_5435_-	ACIAD0183_atpF	Shewana3_4134_-	STM3869_atpF	F0 sector of membrane-bound ATP synthase, subunit b
b3737	*atpE*	COG0636C	Bfl003_atpE	BPEN_003_atpE	BVAF_003_atpE	BUsg003_atpE	BU003_atpE	PputGB1_5436_-	ACIAD0182_atpE	Shewana3_4135_-	STM3870_atpE	F0 sector of membrane-bound ATP synthase, subunit c
b3738	*atpB*	COG0356C	Bfl002_atpB	BPEN_002_atpB	BVAF_002_atpB	BUsg002_atpB	BU002_atpB	PputGB1_5437_-	ACIAD0180_atpB	Shewana3_4136_-	STM3871_atpB	F0 sector of membrane-bound ATP synthase, subunit a
b3741	*mnmG*	COG0445D	Bfl001_gidA	BPEN_001_gidA	BVAF_001_mnmG	BUsg001_gidA	BU001_gidA	PputGB1_5442_-	ACIAD2440_gidA	Shewana3_0002_-	STM3874_gidA	glucose-inhibited cell-division protein
b3771	*ilvD*	COG0129EG	Bfl590_ilvD	BPEN_611_ilvD	BVAF_592_ilvD	BUsg576_ilvD	BU600_ilvD	PputGB1_5178_-	ACIAD1266_ilvD	Shewana3_0358_-	STM3904_ilvD	dihydroxyacid dehydratase
b3774	*ilvC*	COG0059EH	Bfl588_ilvC	BPEN_609_ilvC	BVAF_590_ilvC	BUsg575_ilvC	BU599_ilvC	PputGB1_4676_-	ACIAD3102_ilvC	Shewana3_0355_-	STM3909_ilvC	ketol-acid reductoisomerase, NAD(P)-binding
b3781	*trxA*	COG3118O	Bfl587_trxA	BPEN_608_trxA	BVAF_589_trxA	BUsg573_trxA	BU597_trxA	PputGB1_5276_-	ACIAD3037_trxA	Shewana3_0409_-	STM3915_trxA	thioredoxin 1
b3783	*rho*	COG1158K	Bfl586_rho	BPEN_607_rho	BVAF_588_rho	BUsg572_rho	BU596_rho	PputGB1_5275_rho	ACIAD3038_rho	Shewana3_0408_rho	STM3917_rho	transcription termination factor
b3809	*dapF*	COG0253E	Bfl579_dapF	BPEN_600_dapF	BVAF_581_dapF	BUsg568_dapF	BU589_dapF	PputGB1_5289_dapF	ACIAD2659_dapF	Shewana3_0391_dapF	STM3947_dapF	diaminopimelate epimerase
b3863	*polA*	COG0749L	Bfl619_polA	BPEN_645_polA	BVAF_620_polA	BUsg416_pol	BU431_polA	PputGB1_0138_-	ACIAD3030_polA	Shewana3_4109_-	STM3999_polA	fused DNA polymerase I 5'->3' polymerase/3'->5' exonuclease/5'->3' exonuclease
b3919	*tpiA*	COG0149G	Bfl601_tpiA	BPEN_623_tpiA	BVAF_604_tpiA	BUsg297_tpiA	BU307_tpiA	PputGB1_4716_tpiA	ACIAD0363_tpiA	Shewana3_1026_-	STM4081_tpiA	triosephosphate isomerase
b3924	*fpr*	COG1018C	Bfl600_fpr	BPEN_622_fpr	BVAF_603_fpr	BUsg560_fpr	BU581_fpr	PputGB1_4640_-	ACIAD0747_fpr	Shewana3_3520_-	STM4084_fpr	ferredoxin-NADP reductase
b3936	*rpmE*	COG0254J	Bfl599_rpmE	BPEN_621_rpmE	BVAF_602_rpmE	BUsg556_rpmE	BU577_rpmE	PputGB1_5137_rpmE	ACIAD2210_rpmE	Shewana3_3670_rpmE	STM4096_rpmE	50S ribosomal subunit protein L31
b3941	*metF*	COG0685E	Bfl597_metF	BPEN_619_metF	BVAF_600_metF	BUsg043_metF	BU046_metF	PputGB1_5026_-	ACIAD2283_metF	Shewana3_3612_metF	STM4105_metF	5,10-methylenetetrahydrofolate reductase
b3972	*murB*	COG0812M	Bfl183_murB	BPEN_189_murB	BVAF_184_murB	BUsg042_murB	BU045_murB	PputGB1_1480_murB	ACIAD1945_murB	Shewana3_0182_murB	STM4137_murB	UDP-N-acetylenolpyruvoylglucosamine reductase, FAD-binding
b3981	*secE*	COG0690U	Bfl563_secE	BPEN_583_secE	BVAF_565_secE	BUsg041_secE	BU040_secE	PputGB1_0471_secE	ACIAD0300_secE	Shewana3_0186_secE	STM4147_secE	preprotein translocase membrane subunit
b3982	*nusG*	COG0250K	Bfl562_nusG	BPEN_582_nusG	BVAF_564_nusG	BUsg040_nusG	BU039_nusG	PputGB1_0472_nusG	ACIAD0301_nusG	Shewana3_0187_-	STM4148_nusG	transcription termination factor
b3983	*rplK*	COG0080J	Bfl561_rplK	BPEN_581_rplK	BVAF_563_rplK	BUsg039_rplK	BU038_rplK	PputGB1_0473_rplK	ACIAD0302_rplK	Shewana3_0188_rplK	STM4149_rplK	50S ribosomal subunit protein L11
b3984	*rplA*	COG0081J	Bfl560_rplA	BPEN_580_rplA	BVAF_562_rplA	BUsg038_rplA	BU037_rplA	PputGB1_0474_rplA	ACIAD0304_rplA	Shewana3_0189_rplA	STM4150_rplA	50S ribosomal subunit protein L1
b3985	*rplJ*	COG0244J	Bfl559_rplJ	BPEN_579_rplJ	BVAF_561_rplJ	BUsg037_rplJ	BU036_rplJ	PputGB1_0475_rplJ	ACIAD0305_rplJ	Shewana3_0190_rplJ	STM4151_rplJ	50S ribosomal subunit protein L10
b3986	*rplL*	COG0222J	Bfl558_rplL	BPEN_578_rplL	BVAF_560_rplL	BUsg036_rplL	BU035_rplL	PputGB1_0476_rplL	ACIAD0306_rplL	Shewana3_0191_rplL	STM4152_rplL	50S ribosomal subunit protein L7/L12
b3987	*rpoB*	COG0085K	Bfl557_rpoB	BPEN_577_rpoB	BVAF_559_rpoB	BUsg035_rpoB	BU034_rpoB	PputGB1_0477_rpoB	ACIAD0307_rpoB	Shewana3_0192_rpoB	STM4153_rpoB	RNA polymerase, beta subunit
b3988	*rpoC*	COG0086K	Bfl556_rpoC	BPEN_576_rpoC	BVAF_558_rpoC	BUsg034_rpoC	BU033_rpoC	PputGB1_0478_-	ACIAD0308_rpoC	Shewana3_0193_-	STM4154_rpoC	RNA polymerase, beta prime subunit
b4006	*purH*	COG0138F	Bfl555_purH	BPEN_575_purH	BVAF_557_purH	BUsg032_purH	BU031_purH	PputGB1_4875_purH	ACIAD2447_purH	Shewana3_0441_purH	STM4176_purH	fused IMP cyclohydrolase/phosphoribosylaminoimidazolecarboxamide formyltransferase
b4025	*pgi*	COG0166G	Bfl629_pgi	BPEN_656_pgi	BVAF_629_pgi	BUsg553_pgi	BU573_pgi	PputGB1_4700_pgi	ACIAD0101_pgi	Shewana3_3148_pgi	STM4221_pgi	glucosephosphate isomerase
b4052	*dnaB*	COG0305L	Bfl027_dnaB	BPEN_027_dnaB	BVAF_026_dnaB	BUsg528_dnaB	BU546_dnaB	PputGB1_4930_-	ACIAD2433_dnaB	Shewana3_0713_-	STM4246_dnaB	replicative DNA helicase
b4059	*ssb*	COG0629L	Bfl028_ssb	BPEN_028_ssb	BVAF_027_ssb	BUsg527_ssb	BU545_ssb	PputGB1_0514_-	ACIAD3449_ssb	Shewana3_3590_-	STM4256_ssb	single-stranded DNA-binding protein
b4142	*groS*	COG0234O	Bfl070_groES	BPEN_072_groES	BVAF_070_groES	BUsg018_groES	BU018_groES	PputGB1_4489_groES	ACIAD2839_groES	Shewana3_3568_groES	STM4329_groES	Cpn10 chaperonin GroES, small subunit of GroESL
b4143	*groL*	COG0459O	Bfl071_groEL	BPEN_073_groEL	BVAF_071_groEL	BUsg019_groEL	BU019_groEL	PputGB1_4488_groEL	ACIAD2838_groEL	Shewana3_3567_groEL	STM4330_groEL	Cpn60 chaperonin GroEL, large subunit of GroESL
b4162	*orn*	COG1949A	Bfl075_orn	BPEN_077_orn	BVAF_075_orn	BUsg554_orn	BU574_orn	PputGB1_4954_-	ACIAD3118_orn	Shewana3_0590_-	STM4350_orn	oligoribonuclease
b4177	*purA*	COG0104F	Bfl083_purA	BPEN_086_purA	BVAF_083_purA	BUsg546_purA	BU566_purA	PputGB1_4941_-	ACIAD1258_purA	Shewana3_0695_-	STM4366_purA	adenylosuccinate synthetase
b4200	*rpsF*	COG0360J	Bfl085_rpsF	BPEN_088_rpsF	BVAF_085_rpsF	BUsg544_rpsF	BU564_rpsF	PputGB1_4934_rpsF	ACIAD2430_rpsF	Shewana3_0701_rpsF	STM4391_rpsF	30S ribosomal subunit protein S6
b4202	*rpsR*	COG0238J	Bfl086_rpsR	BPEN_089_rpsR	BVAF_086_rpsR	BUsg543_rpsR	BU563_rpsR	PputGB1_4933_rpsR	ACIAD2431_rpsR	Shewana3_0703_rpsR	STM4393_rpsR	30S ribosomal subunit protein S18
b4203	*rplI*	COG0359J	Bfl087_rplI	BPEN_090_rplI	BVAF_087_rplI	BUsg542_rplI	BU562_rplI	PputGB1_4931_rplI	ACIAD2432_rplI	Shewana3_0704_rplI	STM4394_rplI	50S ribosomal subunit protein L9
b4221	*ytfN*	COG2911S	Bfl090_ytfN	BPEN_093_ytfN	BVAF_090_ytfN	BUsg080_ytfN	BU087_ytfN	PputGB1_2889_-	ACIAD2402_-	Shewana3_1893_-	STM4410_ytfN	conserved protein
b4258	*valS*	COG0525J	Bfl033_valS	BPEN_033_valS	BVAF_032_valS	BUsg354_valS	BU366_valS	PputGB1_0983_valS	ACIAD2950_valS	Shewana3_1131_valS	STM4475_valS	valyl-tRNA synthetase
b4260	*pepA*	COG0260E	Bfl035_pepA	BPEN_035_pepA	BVAF_034_pepA	BUsg355_pepA	BU367_pepA	PputGB1_0986_-	ACIAD0253_pepA	Shewana3_3006_-	STM4477_pepA	multifunctional aminopeptidase A: a cyteinylglycinase, transcription regulator and site-specific recombination factor
