# Supplementary material for: Reduced Selective Constraint in Endosymbionts: Elevation in Radical Amino Acid Replacements Occurs Genome-Wide
Source: PLoS One. 2011 Dec 14;6(12):e28905. doi: 10.1371/journal.pone.0028905 (PMC3237559; doi:10.1371/journal.pone.0028905)
Supplement: Table S3 — Comparison of Dr/Dc between endosymbiotic vs. free-living bacterial species, across seven functional categories. (PDF) [file pone.0028905.s004.pdf]

Supplementary Table S3. Comparison of D_r/D_c between endosymbiotic vs. free-living bacterial species, across seven functional categories.

Two comparisons of endosymbionts vs. free-living bacteria considered: **(a)** *Buchnera APS - Buchnera SG* vs. *Acinetobacter sp. - P. putida,* and **(b)** *Blochmannia floridanus - Blochmannia pennsylvanicus* vs. *E. coli - Shewanella sp*. For each of the seven functional categories considered, D_r/D_c is higher in endosymbionts (pair A) than in related free-living bacteria (pair B). For most comparisons, the lower bound of the 99% confidence interval of [($D_r/D_{c,}$ pair A) / (D_r/D_c, pair B)] (or, the 'elevation index') exceeds one. In addition, sign tests and Wilcoxon tests are highly significant for most comparisons. The few non-significant results were generally those functional categories with relatively few genes and therefore small sample sizes in the statistical tests. The total number of orthologs across the seven functional categories (385) is greater than the 256 considered when functional groups are combined, because some genes are assigned to more than one category and therefore included more than once in the analysis. The values shown here parellel those presented in Table 6 for the combined dataset. (Data presented here for "all" functional categories also appear in Table 6.)

							(D_r/D_c, pair A) / (D_r/D_c, pair B) [a]			sign test				Wilcoxon test	
	pair A	pair B	Functional category	# orthologs used	median D_r/D_c, pair A	median D_r/D_c, pair B	median	mean	(99% confidence interval)	D_r/D_c higher in pair A	D_r/D_c higher in pair B	% of genes with D_r/D_c higher in pair A	p[b]	Chi Square	p[c]
**(a)**	*BuchAPS - BuchSG vs.*	*Acinet.sp - P.putida*	all	256	0.7143	0.5642	1.23	1.29	(1.23 - 1.36)	202	54	78.9%	**1.4E-21**	104.4	**1.70E-24**
			Cell processes	23	0.6993	0.5703	1.17	1.23	(1.04 - 1.41)	18	5	78.3%	**0.0053**	6.2	**0.013**
			Cell structure	73	0.6581	0.5535	1.22	1.25	(1.12 - 1.37)	54	19	74.0%	**2.5E-05**	16.9	**3.93E-05**
			Info transfer	122	0.7120	0.5600	1.25	1.28	(1.20 - 1.37)	93	29	76.2%	**2.6E-09**	43.6	**4.08E-11**
			Metabolism	112	0.7207	0.5815	1.22	1.27	(1.18 - 1.36)	90	22	80.4%	**3.0E-11**	44.2	**2.98E-11**
			Regulation	19	0.7286	0.5695	1.22	1.21	(0.999 - 1.43)	13	6	68.4%	0.084	5.7	**0.017**
			Transport	22	0.6549	0.5618	1.18	1.26	(1.02 - 1.50)	19	3	86.4%	**4.3E-04**	8.2	**0.0042**
			Unclassified	14	0.7565	0.5440	1.45	1.64	(1.03 - 2.24)	12	2	85.7%	**0.0065**	14.5	**1.37E-04**
**(b)**	*Bloch.flor - Bloch.pe vs.*	*E.coli - Shew*.sp.	all	256	0.6552	0.5643	1.17	1.22	(1.17 - 1.28)	185	71	72.3%	**3.4E-13**	60.9	**5.87E-15**
			Cell processes	23	0.5735	0.5697	1.10	1.21	(0.95 - 1.47)	14	9	60.9%	0.20	3.1	0.081
			Cell structure	73	0.6021	0.5645	1.06	1.18	(1.06 - 1.31)	45	28	61.6%	**0.030**	6.6	**0.010**
			Info transfer	122	0.6458	0.5693	1.17	1.21	(1.13 - 1.29)	89	33	73.0%	**2.0E-07**	25.6	**4.11E-07**
			Metabolism	112	0.6739	0.5664	1.16	1.20	(1.12 - 1.28)	81	31	72.3%	**1.3E-06**	26.0	**3.34E-07**
			Regulation	19	0.6135	0.5558	1.20	1.40	(1.09 - 1.71)	14	5	73.7%	**3.2E-02**	7.6	**0.0058**
			Transport	22	0.5859	0.5253	1.07	1.16	(0.97 - 1.35)	15	7	68.2%	0.067	2.7	0.10
			Unclassified	14	0.7152	0.5288	1.41	1.39	(1.06 - 1.72)	11	3	78.6%	**0.029**	8.1	**0.0044**

[a] For each comparison between two genome pairs, the ratio of the two D_r/D_c values is the 'elevation index' described in the text and represented in Figures 3 and 4. The index was calculated for each of the orthologs considered, and then the subsequent calculations (of median, mean, and confidence intervals) were performed.

[b] Significance of the sign test was evaluated using an exact one-sided binomial test of the null hypothesis that D_r/D_c is not higher in the endosymbiont pair. Boldface values reflect significance at the $p<0.05$ level.

[c] Significance of the Wilcoxon test was evaluated using a ChiSquare approximation. Boldface values reflect significance at the $p<0.05$ level.
