# Supplementary material for: Reduced Selective Constraint in Endosymbionts: Elevation in Radical Amino Acid Replacements Occurs Genome-Wide
Source: PLoS One. 2011 Dec 14;6(12):e28905. doi: 10.1371/journal.pone.0028905 (PMC3237559; doi:10.1371/journal.pone.0028905)
Supplement: Table S4 — Amino acid classification schemes used in this study. (PDF) [file pone.0028905.s005.pdf]

Supplementary Table S4. Amino acid classification schemes used in this study. Within each classification scheme, numbers indicate amino acid groups. Changes within a given numerical group are considered conservative, and changes between groups are considered radical. (The specific number used is arbitrary and has to relationship to that group number under a different classification scheme.) Final four columns list some amino acid properties related to the classification schemes.

Amino acid			Radical vs. Conservative classification schemes				Amino acid properties			
			Miyata et al. (MY)	Polarity	Charge	Hanada et al. (HAN)	Volume	Charge	Polarity	Hydropathy
Alanine	Ala	A	1	1	3	1	very small	uncharged	nonpolar	hydrophobic
Proline	Pro	P	1	1	3	1	small	uncharged	nonpolar	neutral
Glycine	Gly	G	1	2	3	1	very small	uncharged	nonpolar	neutral
Serine	Ser	S	1	2	3	1	very small	uncharged	polar	neutral
Threonine	Thr	T	1	2	3	1	small	uncharged	polar	neutral
Asparagine	Asn	N	2	2	3	1	small	uncharged	polar	hydrophilic
Aspartic acid	Asp	D	2	2	1	4	small	neg charged	polar	hydrophilic
Glutamic acid	Glu	E	2	2	1	4	medium	neg charged	polar	hydrophilic
Glutamine	Gln	Q	2	2	3	3	medium	uncharged	polar	hydrophilic
Arginine	Arg	R	3	2	2	3	large	pos charged	polar	hydrophilic
Histidine	His	H	3	2	2	3	medium	pos charged	polar	neutral
Lysine	Lys	K	3	2	2	3	large	pos charged	polar	hydrophilic
Isoleucine	Ile	I	4	1	3	2	large	uncharged	nonpolar	hydrophobic
Leucine	Leu	L	4	1	3	2	large	uncharged	nonpolar	hydrophobic
Methionine	Met	M	4	1	3	2	large	uncharged	nonpolar	hydrophobic
Valine	Val	V	4	1	3	2	medium	uncharged	nonpolar	hydrophobic
Phenylalanine	Phe	F	5	1	3	3	very large	uncharged	nonpolar	hydrophobic
Tryptophan	Trp	W	5	1	3	3	very large	uncharged	nonpolar	hydrophobic
Tyrosine	Tyr	Y	5	2	3	3	very large	uncharged	polar	neutral
Cysteine	Cys	C	6	2	3	1	small	uncharged	nonpolar	hydrophobic
